# TRAP1 expression elicits pro-tumoral functions in macrophages associated to malignant peripheral nerve sheath tumor cells

**DOI:** 10.1186/s13046-025-03525-1

**Published:** 2025-08-28

**Authors:** Francesca Scantamburlo, Alessia Rubini, Margherita Toffanin, Maria Egle Castorina, Francesco Ciscato, Sofia Tomasoni, Paolo Finotti, Ranieri Verin, Valentina Zappulli, Marco Fantuz, Camilla Bean, Andrea Rasola, Ionica Masgras

**Affiliations:** 1 Department of Biomedical Sciences, University of Padova, via Ugo Bassi 58/B, Padova, 35131 Italy; 2 Institute of Neuroscience, National Research Council, via Ugo Bassi 58/B, Padova, 35131 Italy; 3 Department of Comparative Biomedicine and Food Science, University of Padova, viale dell’Università 16, Legnaro, 35020 PD Italy; 4 Department of Veterinary Sciences, University of Pisa, viale delle Piagge 2, Pisa, 56124 Italy; 5 Department of Biology, University of Padova, via Ugo Bassi 58/B, Padova, 35131 Italy; 6 Veneto Institute of Molecular Medicine, via G. Orus 2, Padova, 35129 Italy; 7 Department of Medicine, University of Udine, P.le Kolbe 3, Udine, 33100 Italy

**Keywords:** TRAP1, MPNST, Macrophages, Angiogenesis, HIF-1α

## Abstract

**Background:**

Metabolic adaptations can sustain the pro-neoplastic functions exerted by macrophages in the tumor microenvironment. Malignant peripheral nerve sheath tumors (MPNSTs), aggressive and incurable sarcomas that develop either sporadically or in the context of the genetic syndrome Neurofibromatosis type 1, are highly infiltrated by macrophages, whose contribution to MPNST growth remains poorly characterized. Here, we analyze the role played by the molecular chaperone TRAP1, a regulator of mitochondrial metabolic pathways, in shaping the pro-tumoral activity of macrophages associated to MPNST cells.

**Methods:**

We have studied the phenotypic changes elicited by a MPNST cell-conditioned medium in macrophages with or without TRAP1, and their subsequent ability to support MPNST cell growth and migration and endothelial cell angiogenesis.

**Results:**

The presence of TRAP1 is required in both naive and M2-like macrophages for eliciting phenotypic changes that lead to the acquisition of pro-neoplastic features. TRAP1-expressing macrophages become able to sustain MPNST cell growth and migration and to exert pro-angiogenic properties on endothelial cells through accumulation of the metabolite succinate and the ensuing activation of a HIF-1α-dependent transcriptional program.

**Conclusions:**

Our data provide evidence of a molecular crosstalk between MPNST cellular components, in which soluble factors released by cancer cells drive phenotypic changes in macrophages that in turn enhance pro-tumoral biological routines in both MPNST and endothelial cells. TRAP1-dependent metabolic rewiring in macrophages is mandatory for sustaining this interplay, as a TRAP1-succinate-HIF-1α signaling axis orchestrates their acquisition of tumor-promoting features.

**Graphical abstract:**

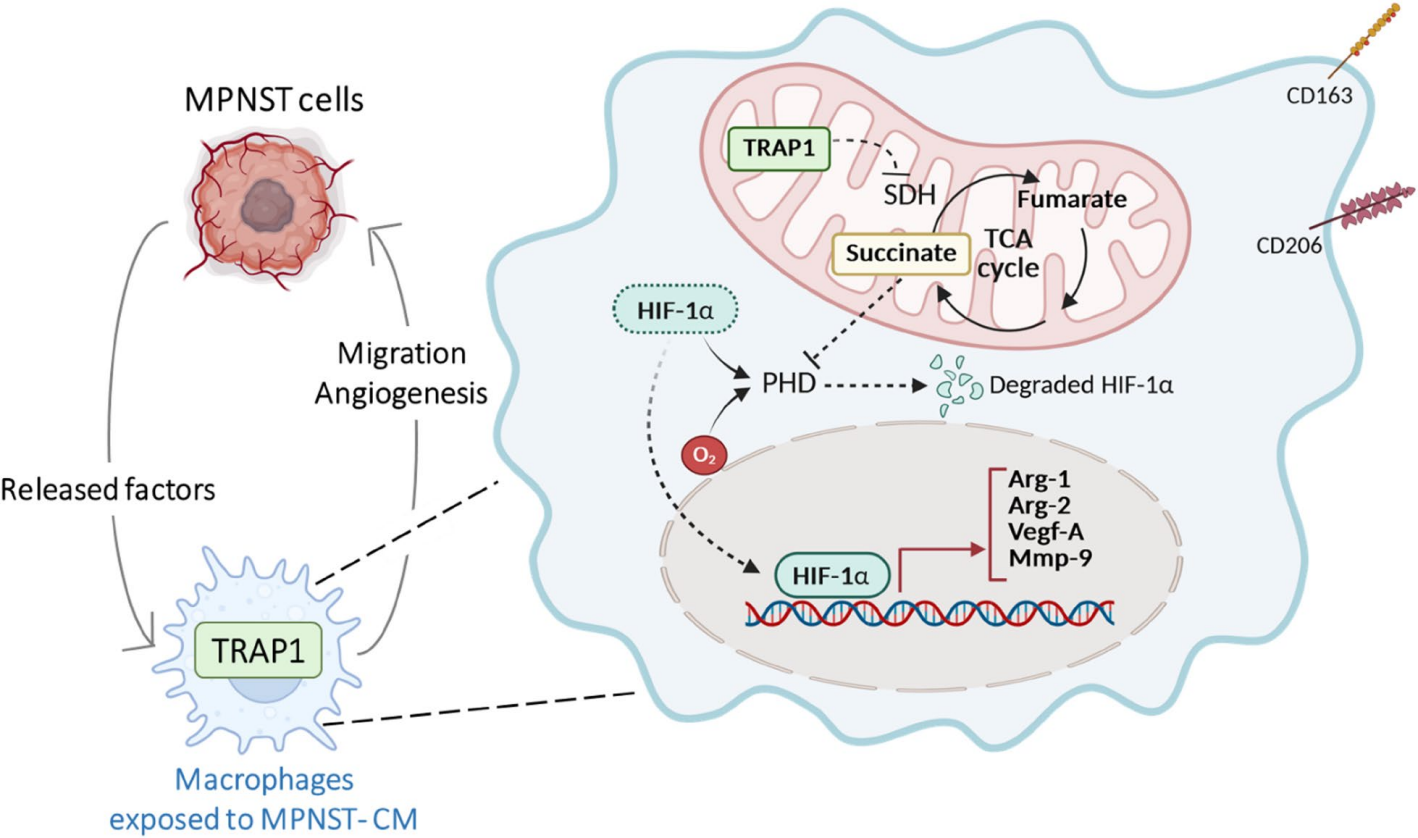

**Supplementary Information:**

The online version contains supplementary material available at 10.1186/s13046-025-03525-1.

## Introduction

Malignant peripheral nerve sheath tumors (MPNSTs) are aggressive cancers that arise from the Schwann cell lineage and develop either sporadically or in patients affected by Neurofibromatosis type 1 (NF1), a genetic disease hallmarked by benign peripheral nerve tumors called neurofibromas [1]. In the plexiform subtype of these tumors, lesions can progress toward the malignant form that constitutes the major cause of death for NF1 patients, as no cure is currently available [2]. Hence, a careful analysis of the determinants of MPNST growth is mandatory to conceive effective therapeutic approaches. In this scenario, the onset of a range of metabolic adaptations during the neoplastic process could offer targetable liabilities. For instance, MPNST cells utilize high amounts of glutamine for biosynthetic purposes, opening the possibility of obtaining anti-tumor effects by inhibition of glutaminase, a key enzyme for glutamine usage [3, 4]. Moreover, we have found that MPNST cells repress oxidative phosphorylation through downregulation of both NADH dehydrogenase and succinate dehydrogenase (SDH) (respiratory complexes I and II, respectively) [5, 6]. A master regulator of this metabolic rewiring is the mitochondrial chaperone TRAP1: by driving succinate accumulation, it elicits the ensuing stabilization of the pro-neoplastic transcription factor HIF-1α [7], and TRAP1 targeting with newly identified and selective inhibitors impairs neoplastic growth of MPNST cells [8].

MPNSTs are endowed with a highly heterotypic microenvironment, and among the several cell types that contribute to their growth macrophages constitute the most abundant population [9] mainly characterized by an anti-inflammatory, M2-like phenotype [10–12]. In the latest years, a flurry of studies has demonstrated that tumor-associated macrophages (TAMs) display heterogeneous features, making their classical partition into pro-inflammatory M1 and anti-inflammatory M2 cells an obsolete oversimplification [13]. Nonetheless, it is believed that the presence of M2-like traits characterizes macrophage subpopulations involved in several pro-neoplastic functions that encompass immune escape, angiogenesis, extracellular matrix remodeling and promotion of cancer cell proliferation, survival and dissemination [14–16]. Therefore, it is of paramount importance to investigate how the crosstalk between cancer cells and TAMs shapes their respective functions in the context of MPNST onset and progression.

Of note, specific features of the tumor microenvironment (TME) can dictate peculiarities in macrophage phenotype and functions. TME cues, including tumor-secreted soluble factors [17], have been identified as instrumental drivers in promoting the pro-tumorigenic features of TAMs, while suppressing their anti-tumorigenic characteristics. Recently, extracellular metabolites have emerged as an important component in shaping the phenotype of macrophages [17], while their metabolic signature, largely determined by mitochondrial functions, contributes to the heterogeneity of TAMs in the TME [18, 19] by defining functional commitments and lineage plasticity [20–22]. For instance, the pro-inflammatory M1 polarization requires succinate oxidation by mitochondria, while SDH inhibition shifts the balance toward M2 specification [23]. Succinate is a crucial metabolite in determining macrophage cell identity [24] and its release by cancer cells with defective SDH prompts evolution of non-committed macrophages (Mφ) into TAMs [25, 26]. Moreover, induction of the enzymes arginase 1 (ARG-1) and glutamine synthetase (GS) plays key roles in TAM pro-tumoral functions. Arginase inhibition blunts the myeloid cell-mediated immune evasion [27], while GS blockade prevents the immunosuppressive and pro-angiogenic state that promotes metastasis of lung cancer cells [28]. These discoveries have built the new concept that metabolic reprogramming of TAMs could constitute an additional method for enhancing their antitumor effects [29].

However, neither the molecular cues that determine the phenotype of macrophage populations, nor their functions have been identified yet in the context of MPNSTs, even if a recent study has revealed that macrophages infiltrating MPNSTs have an immunosuppressive state characterized by high expression of the immune checkpoint inhibitor PD-L1 [30]. These findings suggest that macrophages could act as pro-neoplastic actors in MPNST growth, thus posing this cell population as a candidate target for therapeutic strategies aimed at unleashing antitumor immune responses.

In the present study, we have investigated how association with MPNST cells tunes the phenotypic and functional traits of macrophages. Furthermore, we have studied the role of the metabolic regulator TRAP1 in MPNST-conditioned macrophages, identifying a TRAP1-dependent signaling axis that sustains macrophage pro-tumoral polarization. TRAP1 targeting in macrophages could be exploited for anti-neoplastic purposes in MPNSTs.

## Results

### Exposure to a medium conditioned by MPNST cells polarizes macrophages toward a M2-like phenotype in a TRAP1-dependent manner

TAMs are the preeminent cellular component in the MPNST microenvironment and are often skewed toward a M2-like phenotype. However, it is poorly characterized if they contribute to malignancy and how the molecular crosstalk with cancer cells shapes their features. To address whether MPNST cells contribute to this M2-like macrophage polarization, and whether the mitochondrial chaperone TRAP1 is involved in this process, we established an in vitro system using murine bone marrow-derived macrophages (BMDMs). Monocytes were isolated and differentiated into either unpolarized macrophages (Mφ) or M2-like cells using IL-4 [31] (Supplementary Fig. 1A). Mφ macrophages represent an undifferentiated baseline population that mimics the initial exposure of macrophages to MPNST cells, whereas M2-like macrophages are the main cellular component of the MPNST microenvironment, partially modeling TAMs found in vivo [12]. Induction of the enzyme ARG-1 (Supplementary Fig. 1B) confirmed the IL-4-dependent skewing towards a M2-like phenotype. We then exposed these two populations of macrophages to a MPNST-cell conditioned medium (MPNST-CM) to assess if it is able to elicit phenotypic and functional changes. Moreover, we evaluated the potential role of TRAP1 in tuning the effects of MPNST-CM exposure by comparing Mφ/M2 BMDMs obtained from either wild-type (WT) or TRAP1 knock-out (KO) animals (Supplementary Fig. 1A).

**Fig. 1:**
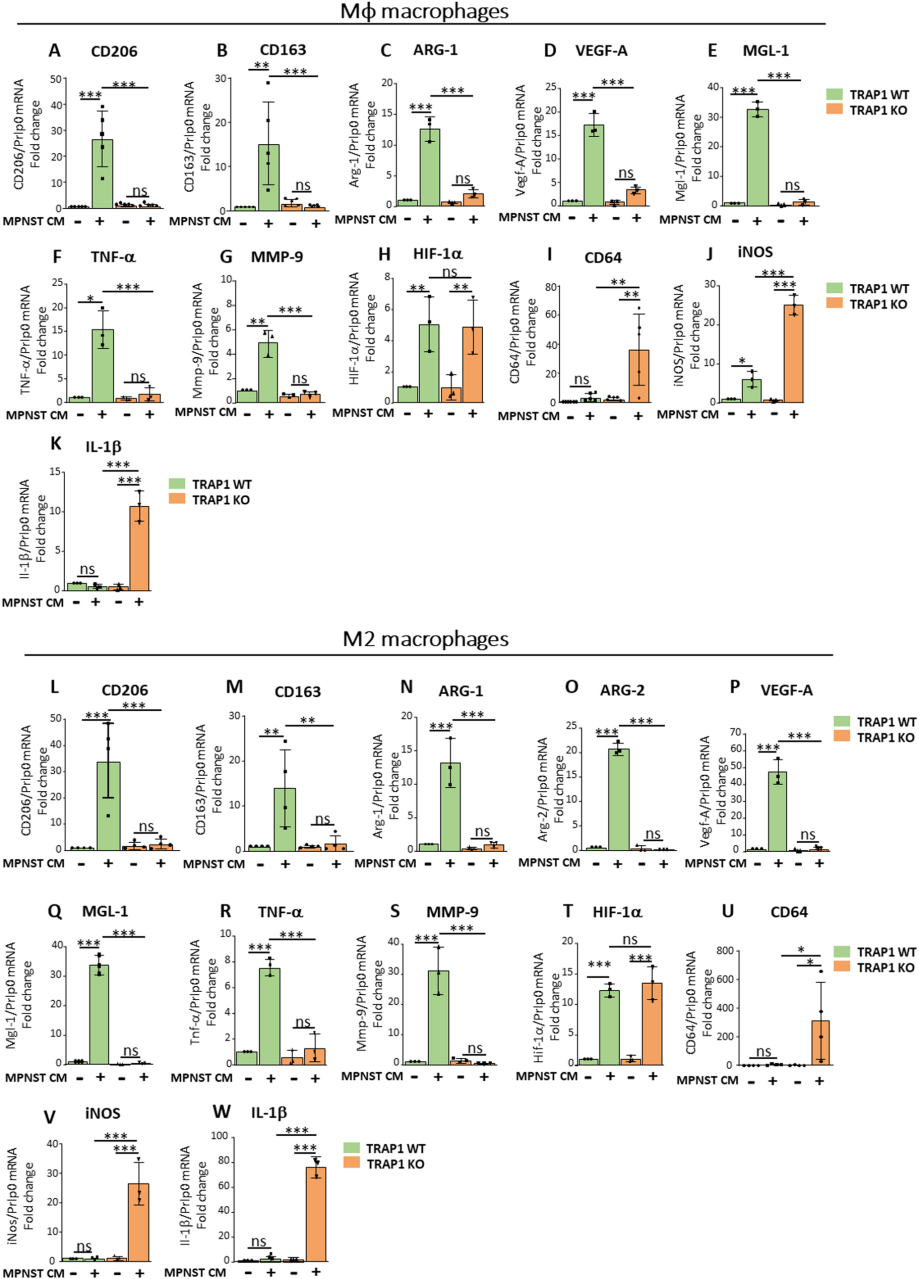
Macrophage exposure to MPNST-CM elicits the expression of M2-like markers in a TRAP1-dependent way. RT-qPCR analysis of (**A**) *CD206*, (**B**) *CD163*, (**C**) *Arg-1*, (**D**) *Vegf-A*, (**E**) *Mgl-1*, (**F**) *Tnf-α*, (**G**) *Mmp-9* and (**H**) *HIF-1α*, (**I**) *CD64*, (**J**) *iNos* and (**K**) *Il-1β * mRNA levels in Mφ macrophages cultured in either MPNST-CM (+) or control medium (-). RT-qPCR analysis of (**L**) *CD206*, (**M**) *CD163*, (**N**) *Arg-1*, (**O**) Arg-2, (**P**) *Vegf-A*, (**Q**) *Mgl-1*, (**R**) *Tnf-α*, (**S**) Mmp-9, (**T**) *HIF-1α*, (**U**) *CD64*, (**V**) *iNos* and (**W**) *IL-1β* mRNA levels in M2 macrophages cultured in MPNST-cell CM (+) or control medium (-). mRNA expression levels were normalized vs. *Prlp0* and FC (fold change) was expressed as mean ± SD of at least three independent experiments. *p < 0.05, **p < 0.01; ***p < 0.001, ****p < 0.0001 with one-way ANOVA test.

We found that Mφ/M2 macrophages exposed to MPNST-CM underwent induction of mRNAs for a set of M2-like TAM markers, i.e. *CD206*,* CD163*,* Arg-1*, *Arg-2*, *Vegf-A*, *Mgl-1*,* TNF-a and Mmp-9*, when compared to cells exposed to a control medium (Fig. 1A-G for Mφ macrophages; Fig. 1L-S for M2 macrophages). Interestingly, upon MPNST conditioning both WT and TRAP1 KO Mφ and M2 macrophages similarly upregulated HIF-1α mRNA, suggesting that the transcriptional regulation of this gene is TRAP1-independent (Fig. 1H, T). TRAP1 ablation in Mφ/M2 macrophages exposed to MPNST-CM strongly prevented the rise in the expression of these markers, while inducing conventional M1-like macrophage mRNAs, *i.e. CD64* (Fig. 1I, U), *iNOS* (Fig. 1J, V) and *IL-1β* (Fig. 1K, W).

Given the established role of TRAP1 in the HIF-1α induction at the post-transcriptional level, and the importance of such induction in driving a cell metabolic reprogramming [7], we examined the expression of HIF-1α itself and of key M2-like macrophage polarization and metabolic markers also at the protein level.

**Fig. 2:**
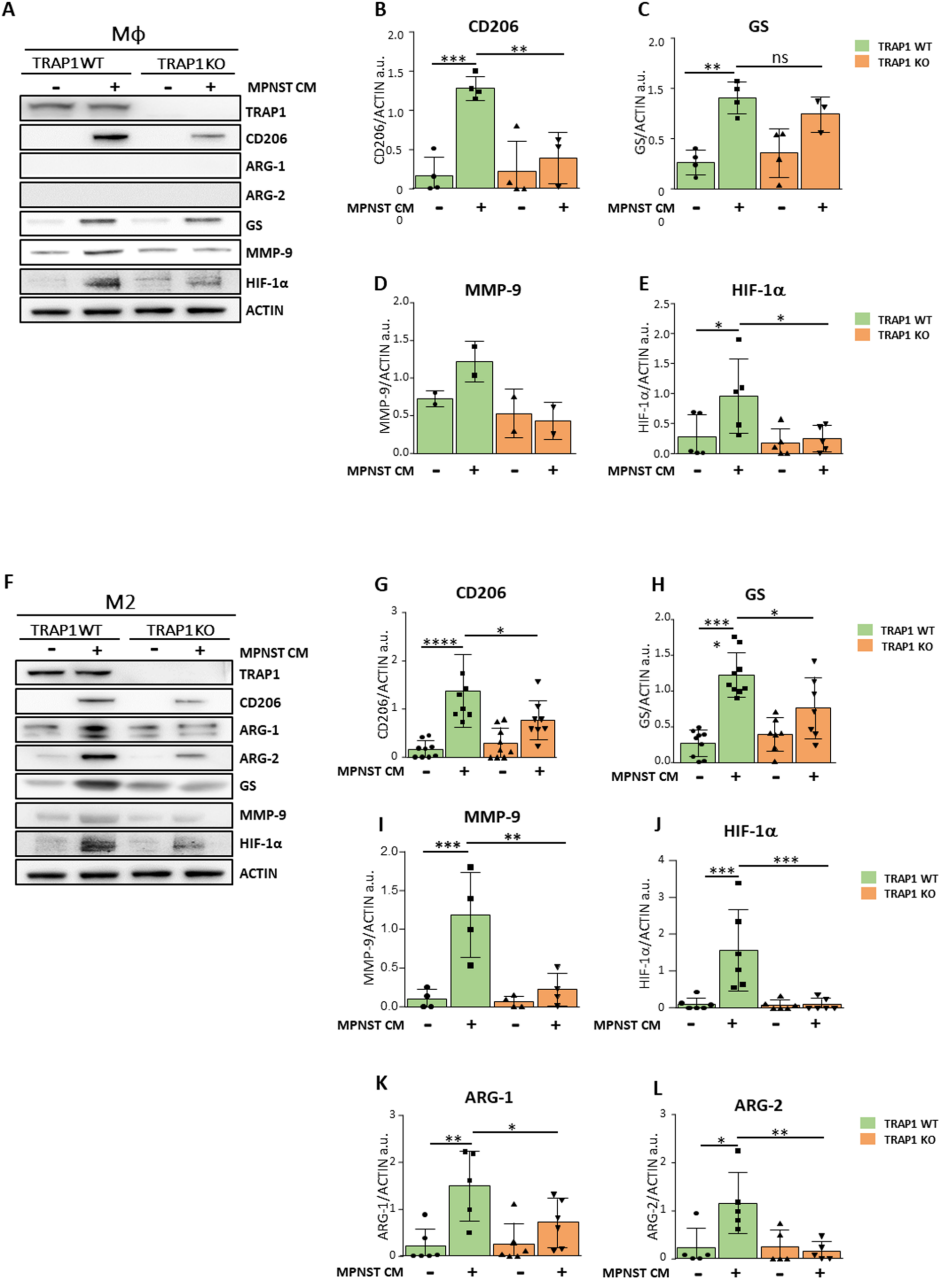
TRAP1 expression modulates the expression of M2-like macrophage markers after exposure to MPNST-CM. (**A**,** F**) Representative Western blot images of changes in protein expression in Mφ (**A**) or M2 (**F**) macrophages cultured for 48 h in MPNST-CM (+) or control medium (-). Quantification of markers in Mφ (**B-E**) and M2 (**G-L**) macrophages. Protein expression levels were normalized vs. actin and expressed as mean ± SD of at least three independent experiments. *p < 0.05, **p < 0.01; ***p < 0.001, ****p < 0.0001 with one-way ANOVA test.

We found that exposure to MPNST-CM induced the mannose receptor CD206 (Fig. 2A, B) and increased protein levels of GS (Fig. 2A, C) in Mφ/M2 macrophages independently of the presence of TRAP1. Instead, TRAP1 was instrumental for upregulating HIF-1α protein (Fig. 2A, E, F, J) and its downstream targets MMP-9 (Fig. 2A, D, F, I) in Mφ/M2 macrophages, and both arginase isoforms (ARG-1/ARG-2) in M2 macrophages (Fig. 2F, K, L). Notably, even though MPNST-CM exposure increases HIF-1α mRNA independently of TRAP1, the presence of the chaperone is required for upregulating HIF-1α protein levels (compare Fig. 1H**/T** with Fig. 2E**/J**). This complex macrophage phenotypic rewiring was similarly induced by their exposure to the medium conditioned by two other MPNST cell models, NPCIS (Supplementary Fig. 2A-J) and cisMPNST cells (Supplementary Fig. 2K-T).

Taken together, these data indicate that MPNST cells have the ability to activate both Mφ and M2 macrophages through the release of secreted factors, and that the mitochondrial chaperone TRAP1 enhances the induction of metabolic enzymes (i.e. ARG-1 and ARG-2), of the transcription factor HIF-1α and of its target genes MMP-9 and VEGF-A, which are known to be involved in tumor invasion and angiogenesis.

### Macrophages exposed to MPNST-CM sustain cancer cell growth and migration in a TRAP1-dependent fashion

**Fig. 3:**
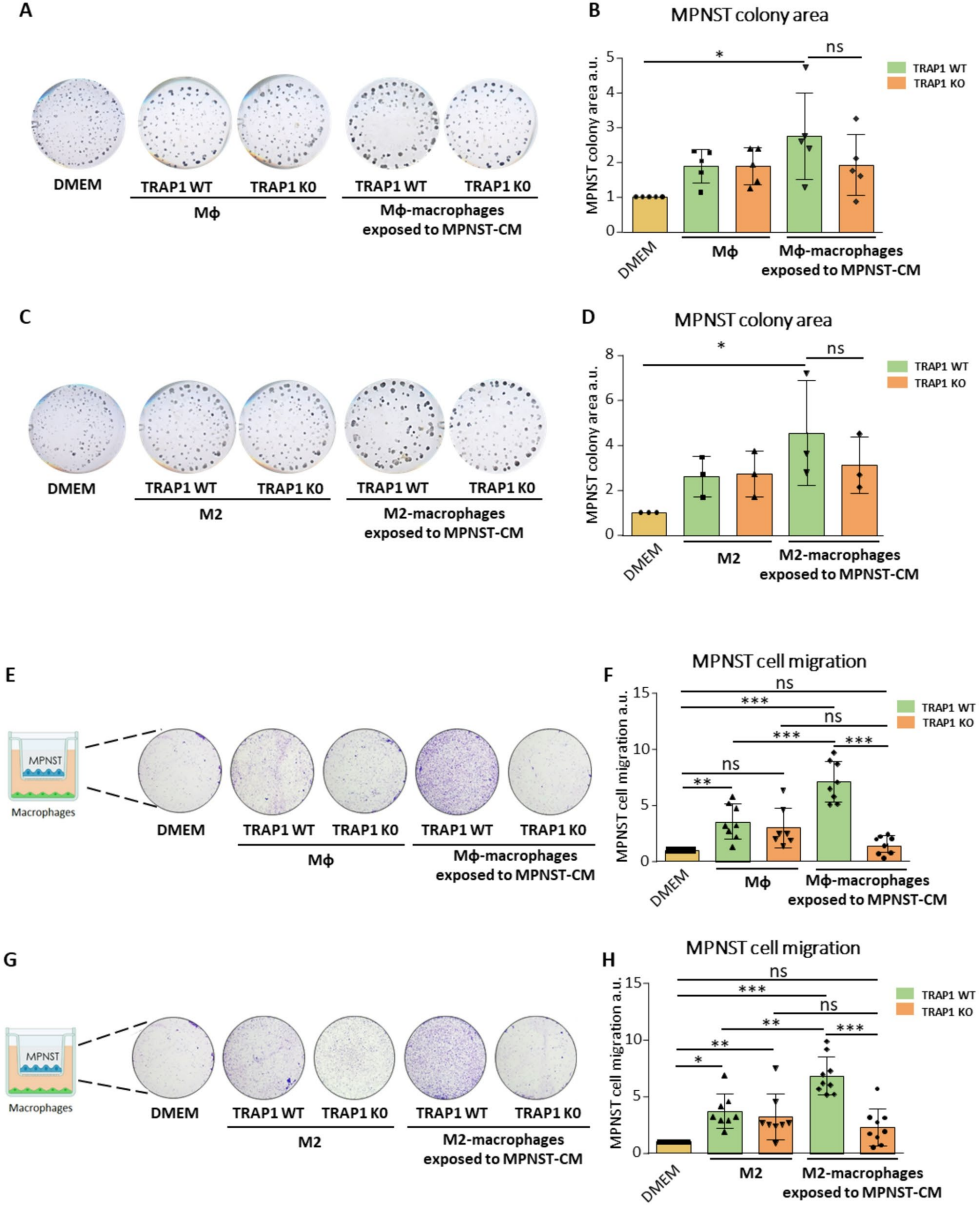
Exposure to MPNST-CM and TRAP1 expression are required for the acquisition of pro-tumorigenic features by macrophages. Representative images (**A**,** C**) and quantification of colony formation by MPNST cells co-cultured with macrophages. Representative images **(E**,** G**) and quantification of migration after co-culturing MPNST cells and macrophages. MPNST conditioned Mφ/M2 macrophages: BMDM exposed to MPNST-CM following the protocol of Supplementary Fig. 1A. In all experiments, MPNST cells plated in DMEM were used as a control. Data are reported as the FC mean ± SD of at least three independent experiments and normalized vs. the control. *p < 0.05, **p < 0.01; ***p < 0.001, ****p < 0.0001 with a one-way ANOVA test.

Therefore, we investigated whether macrophages could tune key cancer cell features upon exposure to MPNST-CM, and if TRAP1 presence could influence their pro-tumoral properties. First, we assessed the effect of macrophages on colony formation of MPNST cells by co-culturing them in a 3D Matrigel matrix. In this setup, MPNST cells were co-cultured in Matrigel with either WT or TRAP1 KO Mφ/ M2 macrophages pre-exposed or not to the MPNST-CM for 48 h; no CM was further added during the colony formation experiment. As a negative control, MPNST cells were grown in Matrigel without macrophages. We found that only TRAP1-expressing macrophages exposed to MPNST-CM markedly increased colony formation by MPNST cells, even though a trend toward the upregulation of colonies was measurable in all co-culture conditions without reaching statistical significance (Fig. 3A-D). We also tested if soluble factors secreted by macrophages could influence MPNST cell motility by co-culture experiments in Boyden chambers. For these experiments, Mφ and M2 macrophages were placed in the lower chamber and exposed to MPNST-CM for 48 h, after which the MPNST-CM was replaced by standard medium for 24 h to allow its enrichment with factors derived from the plated macrophages. Thereafter, MPNST cells were seeded in the upper chamber without adding any further MPNST-CM during the co-culture phase. As a negative control, MPNST cells were plated without macrophages in the lower chamber. We observed that the presence of Mφ/M2 macrophages induced cancer cell migration, and this was maximally upregulated by TRAP1-expressing macrophages exposed to MPNST-CM. In contrast, TRAP1-deficient macrophages failed to support MPNST cell migration (Fig. 3E-H).

The ability of TRAP1-expressing macrophages to sustain cancer cell migration after exposure to MPNST-CM was also confirmed using media conditioned by two additional MPNST cell models (Supplementary Fig. 3A-B).

These data indicate that the Mφ and M2 macrophages exposed to the MPNST-CM acquire important pro-neoplastic functions, as they elicit both colony formation and migration of MPNST cells, and that TRAP1 ablation abrogates the acquisition of these tumorigenic features.

### Macrophages exposed to MPNST-CM promote angiogenesis in a TRAP1-dependent way

A prominent feature of M2-like TAMs is promotion of angiogenesis in different cancer types, thereby significantly contributing to tumor growth and dissemination [14]. Hence, we evaluated whether macrophages can elicit an angiogenic response, if this is modulated by MPNST-CM exposure and the role of TRAP1 in this process. We first performed an endothelial cell tube formation assay by measuring the number and length of branches formed by SVEC endothelial cells when cultured in the presence of a macrophage-conditioned medium (MCM). Experimental conditions included SVEC cells exposed to the culture medium (negative control), to the MPNST-CM and to the MCM from macrophages either previously exposed to MPNST-CM or not.

**Fig. 4:**
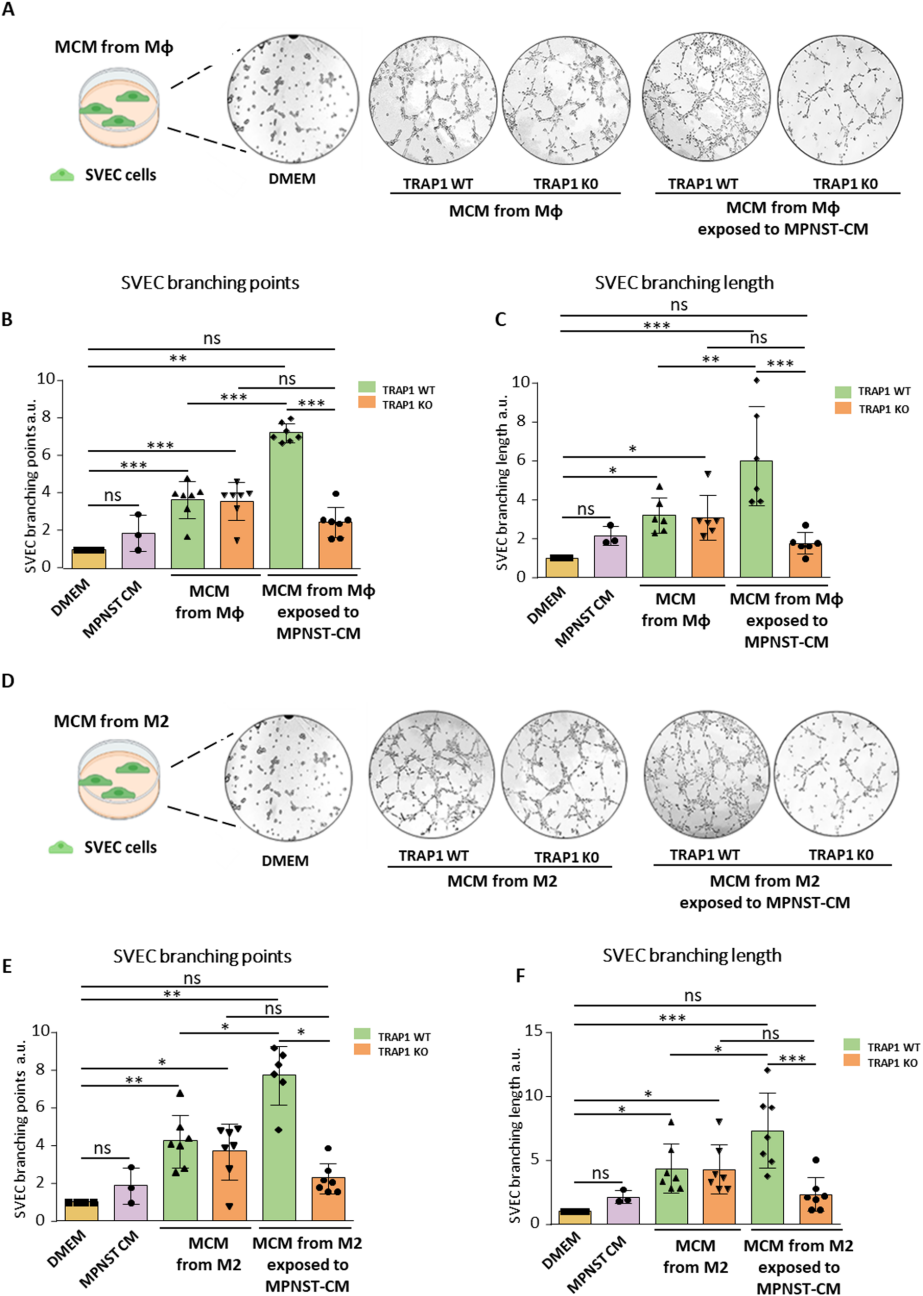
Macrophage promotion of endothelial tube formation is increased by their exposure to MPNST-CM in a TRAP1-dependent way. (**A**,** D**) Representative scheme, images and quantification of branching points (**B**,** E**) and branching length (**C**,** F**) of SVEC cells exposed to DMEM (negative control), to the MPNST-CM and to the MCM obtained from Mφ or M2 cells, or from Mφ/M2 cells previously exposed to MPNST-CM. Data are reported as FC mean ± SD of at least three independent experiments and normalized vs. the control. *p < 0.05, **p < 0.01; ***p < 0.001, ****p < 0.0001 with a one-way ANOVA test.

We found that a 4 h exposure to MPNST-CM per se did not induce any branching of SVEC cells, while MCMs from both Mφ and M2 cells significantly increased both number (branching points) and length of SVEC branches compared to SVECs grown in control medium, independently of TRAP1 expression (Fig. 4). Endothelial tube formation was further boosted by MCMs from Mφ/M2 cells previously exposed to MPNST-CM, but only if these macrophages expressed TRAP1. (Fig. 4).

The induction of pro-angiogenic features of TRAP1-expressing macrophages exposed to MPNST-CM was also confirmed by employing media conditioned by two additional MPNST cell lines, NPCIS cells (Supplementary Fig. 3C-D) and cisMPNST cells (Supplementary Fig. 3E-F).

**Fig. 5:**
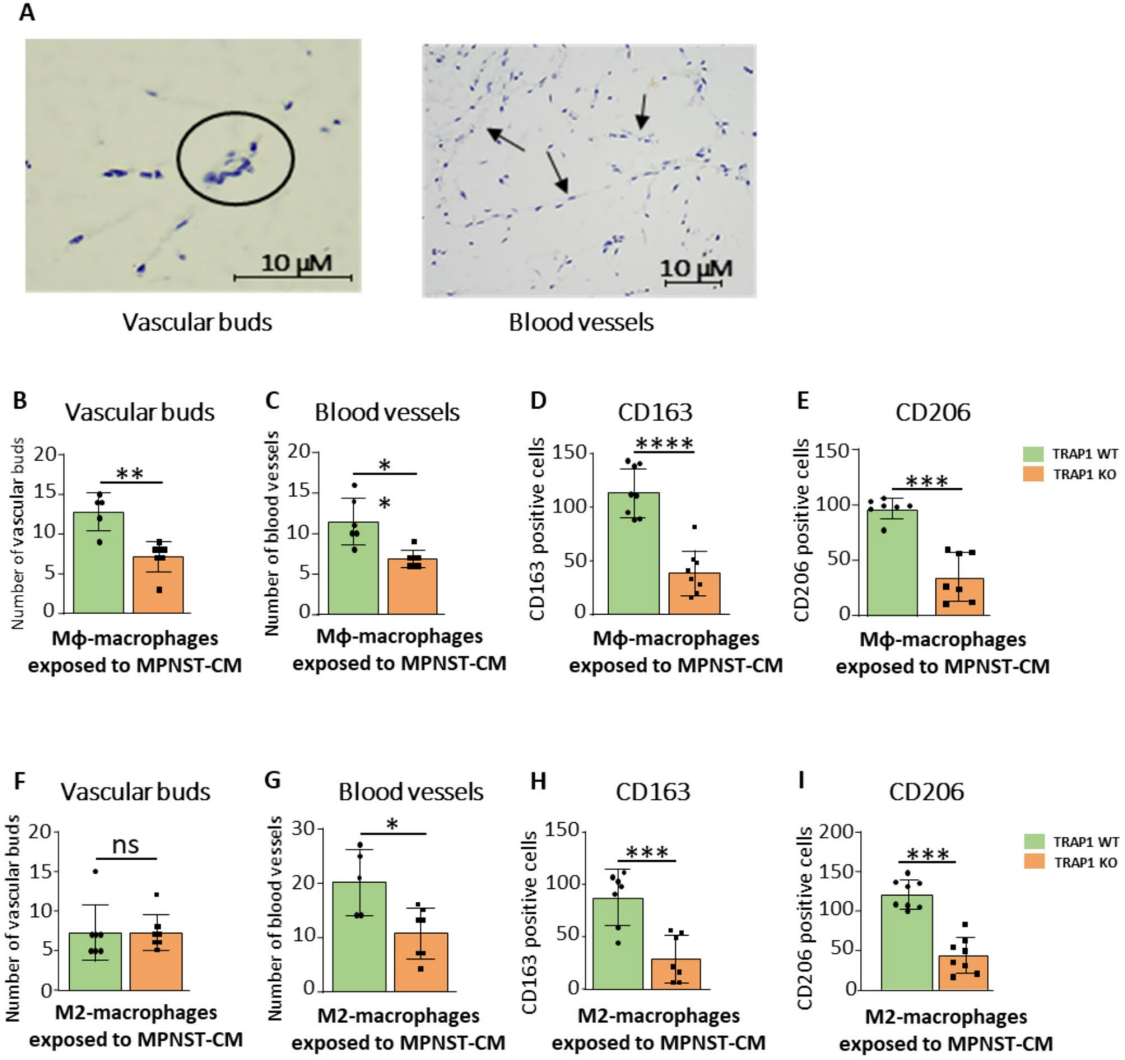
TRAP1 expression increases the angiogenesis promoted by macrophages exposed to MPNST-CM. (**A**) Hematoxylin and eosin staining (H&E) and quantification (**B**,** C**,** F**,** G**) of newly formed vascular buds and blood vessels after injection of macrophage-containing Matrigel plugs. Quantification of cells positive for CD163 **(D**,** H**) and CD206 **(E**,** I**) by IHC inspections. Cells were counted in 10 fields of Matrigel plugs at a 40X magnification. Absolute cell numbers are reported as mean ± SD of at least five experiments. *p < 0.05, **p < 0.01; ***p < 0.001, ****p < 0.0001 with a one-way ANOVA test.

We next investigated whether Mφ/M2 macrophages exposed to MPNST-CM could also sustain angiogenesis in vivo, and the role played by TRAP1 in this process. For this purpose, we evaluated the number of vascular buds and blood vessels after injecting subcutaneously into nude mice either WT or TRAP1 KO cells embedded in a Matrigel matrix (Fig. 5A and Supplementary Fig. 4). We found that all macrophages elicited angiogenesis (Fig. 5B, C, F, G), and the presence of TRAP1 increased the formation of blood vessels, with the highest effect observed in mice injected with M2-derived macrophages (compare the number of blood vessels in Fig. 5C and G). Conversely, the number of vascular buds was higher in mice injected with Mφ vs. M2 cells, and specifically in the presence of TRAP1 (compare Fig. 5B and F). As vascular bud formation is a pre-requisite for the generation of vessels, these data suggest that the angiogenesis process is more rapid in the presence of M2 than of Mφ macrophages. Of note, macrophage infiltration, as measured by IBA-1 staining, was higher in the presence of TRAP1, with a minimal number of M1-like, iNOS^+^ cells and a high proportion of M2-like cells, stained by CD163 and CD206 markers (Fig. 5D, E, H **and I**, Supplementary Fig. 5).

These findings support a model by which endothelial cells co-cultured in the presence of MCM induce the angiogenic process. This effect is further promoted if macrophages had been exposed to MPNST-CM, indicating that MPNST cells are able to boost a pro-angiogenic phenotype in neighboring macrophages. The absence of TRAP1 does not display any effect on the pro-angiogenic potential of Mφ/M2 macrophages, but it prevents the upregulation of this pro-tumoral function prompted by conditioning Mφ/M2 macrophages with MPNST-CM.

Collectively, these findings demonstrate that both Mφ and M2 macrophages exposed to the MPNST-CM enhance pro-angiogenic features in a TRAP1 regulated manner. While the in vivo experiment further suggests that TRAP1 expression is important for increasing the number of macrophages, favoring either their recruitment or viability, the in vitro assay indicates that these macrophages stimulate endothelial cells through the release of soluble factors.

### TRAP1 expression in MPNST-conditioned macrophages induces a pro-angiogenic signature

**Fig. 6:**
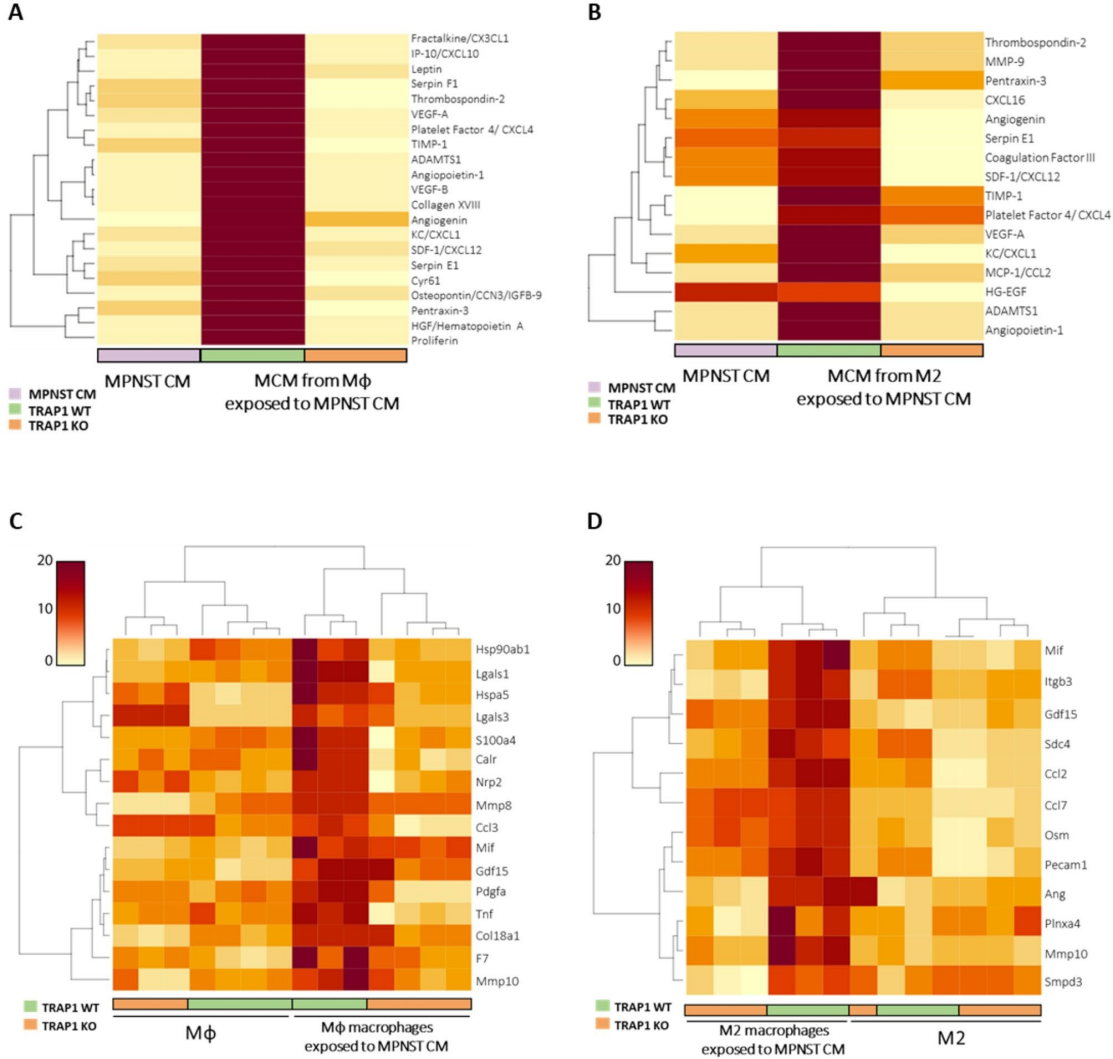
TRAP1 expression is required to establish a pro-angiogenic phenotype in Mφ/M2 cells exposed to MPNST-CM. (**A**,** B**) Heatmaps representing the quantification of the differentially expressed secreted pro-angiogenic factors examined in MPNST-CM and in the MCM of TRAP1 WT and KO Mφ (**A**) and M2 (**B**) cells exposed to cancer cell medium. Data are representative of a pool of three biological replicates and are displayed according to a red-to-yellow color scale. (**C**,** D**) Heatmap of top DEGs filtered for a list of annotated angiogenesis-related, soluble factors in MPNST-conditioned TRAP1 WT and KO Mφ (**C**) and M2 (**D**) cells. Gene expression values are displayed as changes with respect to median expression levels according to a red to yellow color scale. Rows are scaled to better appreciate the differences and the dendrograms show unsupervised hierarchical clustering.

We used a proteome profiler assay tailored for angiogenesis in order to investigate the pro-angiogenic factors released by macrophages exposed to MPNST-CM. On the one hand, we compared the culture media collected from MPNST cells (MPNST-CM) with those from TRAP1-expressing and TRAP1 KO Mφ cells exposed to MPNST-CM; on the other hand, we performed the same comparison, but using M2 instead of Mφ cells. We found that MPNST-CM per se did not contain high levels of any angiogenic factor tested, but both Mφ and M2 cells acquired a pro-angiogenic signature after exposure to MPNST-CM, provided that they expressed TRAP1. Indeed, cells where TRAP1 had been ablated had a profile of released proteins that was almost superimposable to that of MPNST-CM (Fig. 6A-B).

In more detail, the presence of TRAP1 elicited the release from Mφ cells exposed to MPNST-CM of a set of pro-angiogenic chemokines (CX3CL-1, CXCL-10, CXCL-4, CXCL-1 and CXCL-12), pro-angiogenic growth factors and metalloproteinases (VEGF-A, VEGF-B, Angiopoietin-1, HGF, ADAMTS1 and TIMP-1), and regulatory pro-angiogenic mediators (Leptin, Serpin F1, Thrombospondin-2, Collagen XVIII, Angiogenin, Serpin E1, Cyr61, Osteopontin/CCN-3/IGFB-9, Pentraxin-3 and Proliferin) (Fig. 6A). We observed a similar profile for TRAP1-expressing M2 cells, including the pro-angiogenic chemokines CXCL-16, SDF-1/CXCL12, Platelet Factor 4/CXCL-4, KC/CXCL-1 and MCP-1/CCL-2; the pro-angiogenic growth factors and metalloproteinases VEGF-A, HG-EGF, MMP-9, ADAMTS1 and TIMP-1; the pro-angiogenic regulatory factors Thrombospondin-2, Pentraxin-3, Angiogenin, Serpin E1, Coagulation Factor III and Angiopoietin-1 (Fig. 6B); notably, 11 out of these 16 induced proteins were shared between TRAP1-expressing M2 and Mφ macrophages.

We then analyzed both WT and TRAP1 KO Mφ/M2 cells exposed to MPNST-CM at the transcriptomic level with a bulk RNA-seq approach. A preliminary selection was performed to confidently identify secreted factors, employing a previously published bioinformatic pipeline [32]. Analysis of the transcripts included in the “Hallmark-Angiogenesis” collection [33] and encoding for soluble molecules revealed the up-regulation of a set of pro-angiogenic factors in comparison to Mφ/M2 macrophages maintained in control media. These factors encompassed growth factors, cytokines and metalloproteinases such as MMP-8, MMP-10, CCL-3, PDGF-A and TNF in Mφ macrophages exposed to MPNST-CM (Fig. 6C), and ITGB-3, CCL-2, CCL-7, ANG and MMP-10 in M2 conditioned macrophages (Fig. 6D). In keeping with data on released proteins, this pro-angiogenic transcriptomic signature was abolished by TRAP1 ablation in both Mφ and M2 cells (Fig. 6C, D).

Overall, these data indicate that TRAP1 expression profoundly affects the transcriptional landscape of macrophages upon stimulation with the medium conditioned by MPNST cells. We therefore investigated a possible mechanistic link between TRAP1 activity as a regulator of mitochondrial bioenergetics [7] and the transcriptional output of macrophages.

### TRAP1 activates a succinate-HIF-1α signaling pathway in macrophages exposed to MPNST-CM

We reasoned that TRAP1 could tune transcription by regulating cell redox equilibrium, as it acts as a ROS scavenger in different tumor types [34]. Thus, we investigated the potential impact of TRAP1 expression on ROS management in Mφ and M2 macrophages exposed to MPNST-CM. However, we could not detect any difference when we compared intracellular ROS levels in TRAP1 WT and KO Mφ/M2 macrophages exposed or not to MPNST-CM (Supplementary Fig. 6A-B).

We have previously shown that TRAP1 increases intracellular levels of succinate as a result of its inhibitory effect on succinate dehydrogenase (SDH) [7], and TRAP1-dependent succinate accumulation can induce the stabilization of HIF-1α [5, 7], unleashing its transcriptional activity. Conversely, an increase in alpha-ketoglutarate (α-KG) levels prompts the proteasomal degradation of HIF-1a [35].

**Fig. 7:**
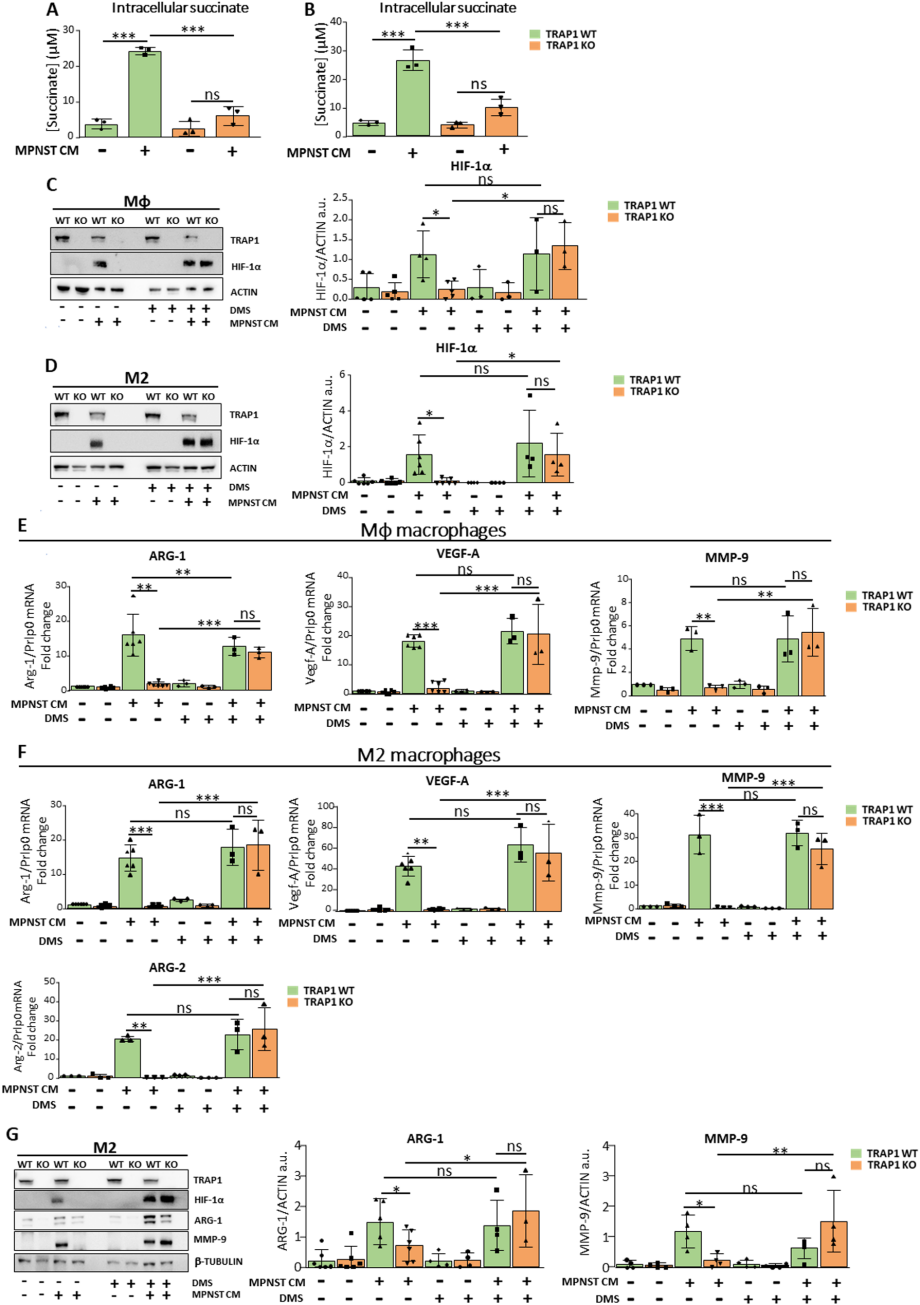
TRAP1 expression elicits a succinate-dependent induction of HIF-1α transcriptional activity after macrophage exposure to MPNST-CM. (**A**,** B**) Quantification of intracellular succinate levels in Mφ (**A**) or M2 (**B**) cells. Data are reported as mean ± SD of three independent experiments. (**C-D**) Western blot analysis and quantification of HIF-1α protein levels in Mφ (**C**) and M2 (**D**) macrophages cultured for 48 h in MPNST- CM (+) or control medium (-), with or without 1 mM DMS. (**E**,** F**) RT-qPCR analysis of *Arg-1*, *Vegf-A*,* Mmp-9*,* Arg-2* mRNA levels in Mφ (**E**) or M2 (**F**) macrophages cultured in MPNST-CM (+) or control medium (-) with or without 1 mM DMS. FC are reported as mean ± SD of at least three independent experiments. (**G**) Western blot analysis and quantification of ARG-1 and MMP-9 expression in M2 macrophages cultured for 48 h in MPNST-cell CM (+) or control medium (-), with or without 1 mM DMS. Protein levels were normalized vs. actin and expressed as mean ± SD of at least three independent experiments. *p < 0.05, **p < 0.01; ***p < 0.001, ****p < 0.0001 with a one-way ANOVA test.

Therefore, we analyzed intracellular succinate levels and HIF-1α protein expression, finding that both were upregulated in Mφ/M2 TRAP1-expressing cells after exposure to MPNST-CM, whereas no changes were detectable in cells where TRAP1 was absent (Fig. 7A-B for succinate and Fig. 7C-D for HIF-1α). Instead, in line with a lack of HIF-1α stabilization, Mφ/M2 TRAP1-deficient macrophages exposed to MPNST-CM increased intracellular levels of α-KG (Supplementary Fig. 7A-B).

We further investigated the presence of a TRAP1-succinate-HIF-1α signaling axis by using the cell-permeable succinate analogue dimethyl-succinate (DMS). Our data indicate that DMS stabilizes HIF-1α in TRAP1-deficient, Mφ/M2 macrophages exposed to MPNST-CM (Fig. 7C-D).

We then assessed the HIF-1α activity, finding a perfect match between HIF-1α stabilization and transcriptional induction of a set of HIF-1α target genes (*Arg-1*, *Vegf-A* and *Mmp-9*) in Mφ/M2 macrophages exposed to MPNST-CM and either expressing TRAP1 or treated with DMS (Fig. 7E-F); in addition, M2 cells increased the mRNA levels of *Arg-2*, which was undetectable in Mφ macrophages (Fig. 7F). In keeping with their transcriptional regulation, the protein expression of both ARG-1 and MMP-9 was boosted in M2 macrophages exposed to MPNST-CM and either expressing TRAP1 or treated with DMS (Fig. 7G).

We next employed dimethyl-α-KG (DM-α-KG), a cell-permeable α-KG analogue that could elicit HIF-1α proteasomal degradation [35]. Our results show that treatment of M2 TRAP1 WT macrophages with MPNST-CM in the presence of 1 mM DM-α-KG significantly decreased the expression of canonical HIF-1α target genes (Supplementary Fig. 7C-F).

These findings strongly support the importance of the TRAP1-mediated succinate/α-KG balance in regulating HIF-1α activity in macrophages exposed to MPNST-CM.

### Succinate confers pro-neoplastic functions to TRAP1-deficient macrophages exposed to MPNST-CM

**Fig. 8:**
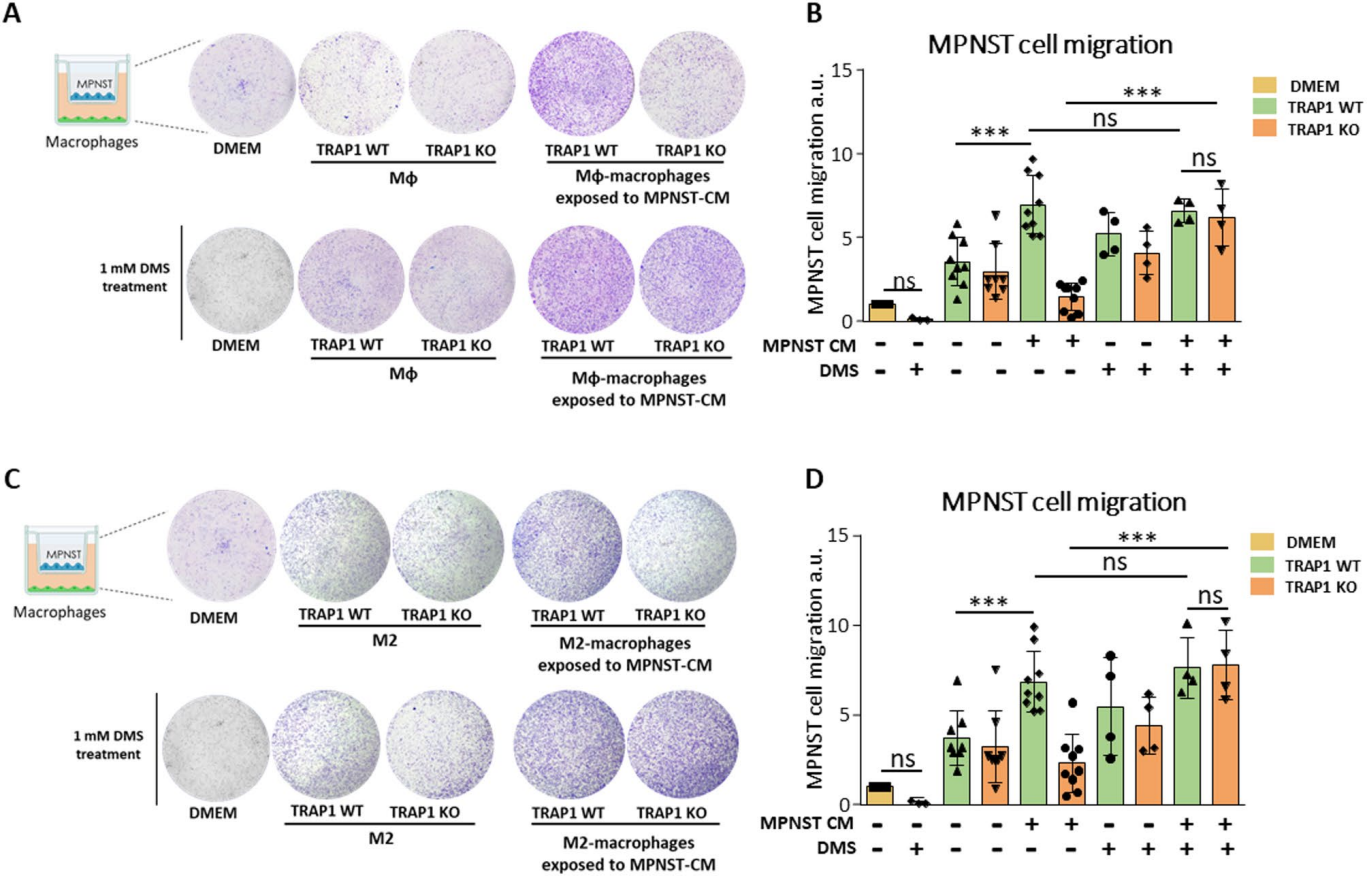
DMS treatment selectively increases the ability of TRAP1 KO Mφ- and M2-conditioned macrophages to induce MPNST cell migration. Representative Boyden chamber experiments (**A**,** C**) and quantification of MPNST cell migration (**B**,** D**). MPNST cells were co-cultured with either Mφ (**A**,** B**) or M2 (**C**,** D**) cells; where indicated, macrophages were pre-conditioned with MPNST-CM and/or treated with 1 mM DMS. Data are reported as mean ± SD of at least four independent experiments. *p < 0.05, **p < 0.01; ***p < 0.001, ****p < 0.0001 with a one-way ANOVA test.

We asked whether exposure to MPNST-CM in the presence of DMS could render TRAP1 KO Mφ/M2 macrophages capable of inducing motility of cancer cells, thus mimicking the effect of the conditioned medium on TRAP1 WT cells. By using a Boyden chamber assay (Fig. 8), we found that DMS markedly increased the induction of MPNST cell motility by TRAP1 KO macrophages, making it reach the same level observed when cancer cells were co-cultured with TRAP1 WT macrophages (Fig. 8).

**Fig. 9:**
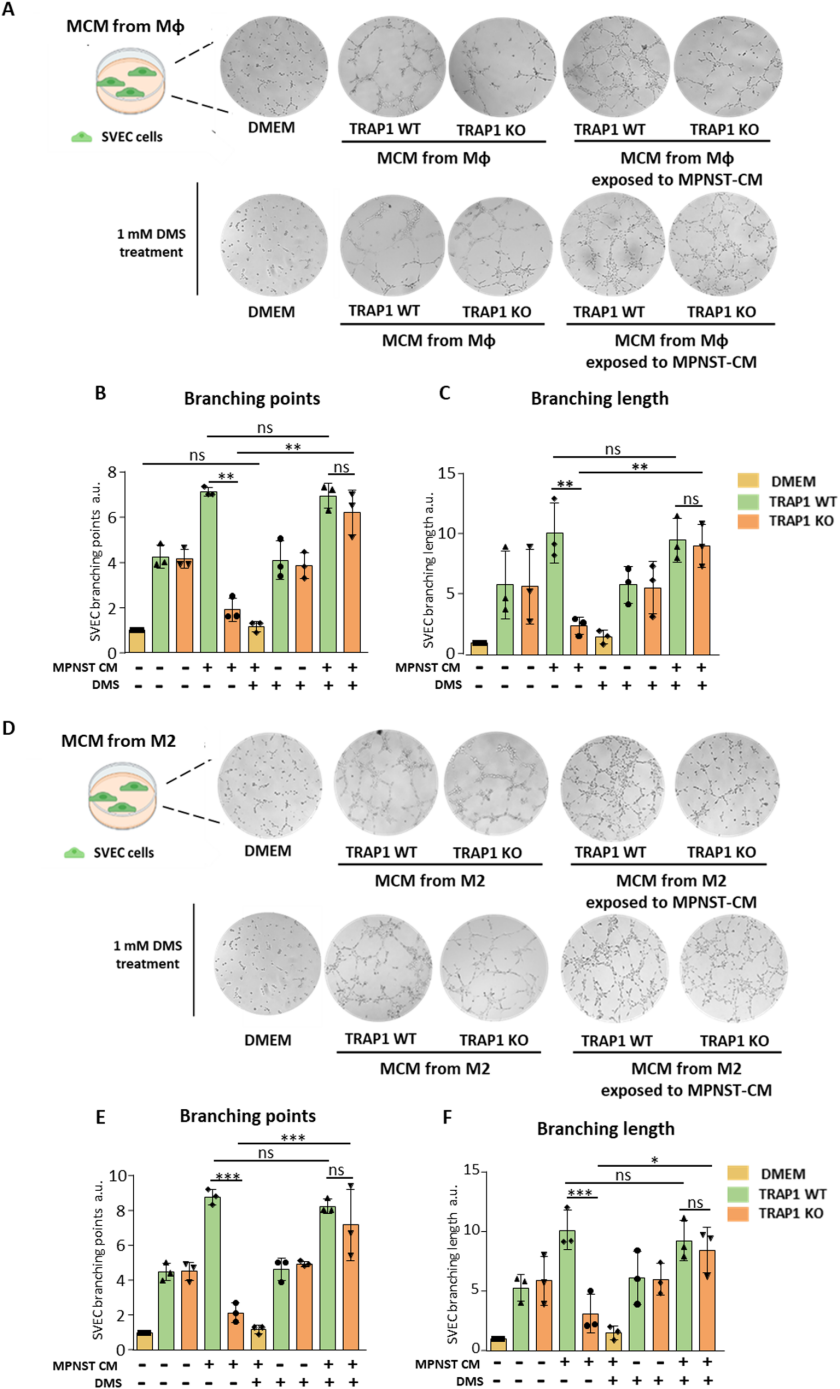
DMS treatment rescues TRAP1 KO Mφ- and M2-conditioned macrophages ability to induce in vitro angiogenesis. (**A**) Representative scheme, images and quantification of (**B**) branching points and (**C**) branching length of SVEC cells cultured in MCM from either Mφ macrophages or Mφ macrophages exposed to MPNST-CM and/or treated with 1 mM DMS. (**D**) Representative scheme, images and quantification of (**E**) branching points and (**F**) branching length measurements on SVEC cells cultured in MCM from either M2 macrophages or M2 macrophages exposed to MPNST-CM and/or treated with 1 mM DMS. DMEM was used as a control. The number of branches (branching points) and branching length of SVEC cells were measured using ImageJ software and expressed as FC compared to control (DMEM) in the form of a bar graph. Data are reported as mean ± SD of four independent experiments. *p < 0.05, **p < 0.01; ***p < 0.001, ****p < 0.0001 with a one-way ANOVA test.

We also studied whether DMS administration could upregulate angiogenesis induction by TRAP1 KO macrophages. Similarly to what observed in the migration assay, DMS supplementation markedly affected the pro-angiogenic phenotype of TRAP1 KO Mφ/M2 macrophages, strongly increasing it to an extent comparable to that of WT cells (Fig. 9).

Collectively, these results are in accord with the presence of a TRAP1- and succinate-dependent modulation of macrophage functions, which is responsible for secreting pro-invasive and pro-angiogenic factors once macrophages interact with MPNST cells.

## Materials and methods

### Cell culture and conditioned medium

The sMPNST and cisMPNST cell models were kindly provided by Dr. Lu Q. Le (Charlottesville, VA, USA), NPCIS cell line was a gift from Dr. Verena Staedtke (Johns Hopkins University, USA) and the mouse fibroblast L-cell strain 929 (L929) was obtained from Dr. Elisa Greggio (University of Padova, Italy). The sMPNST (SKP-derived MPNST) cell line is a murine MPNST cell line derived from tumors established by autologous transplantation of skin-derived precursor cells (SKPs) lacking both Nf1 and p53. Such SKPs have been demonstrated to consistently give rise to tumors with all the histological hallmarks of human MPNSTs [36]. NPCIS and cisMPNST cell lines were derived from tumors developed by the NPcis (B6;129S2-Trp53^tm1Tyj^ Nf1^tm1Tyj^/J) transgenic mice [37] and used as MPNST cell models [4, 36].

All cell lines were grown in Dulbecco’s modified Eagle’s medium (DMEM) supplemented with 10% heat-inactivated fetal bovine serum (FBS), 3.97 mM glutamine, 1 mM sodium pyruvate and 0.1 mM penicillin and streptomycin (P/S), at 37 °C in a humidified atmosphere containing 5% CO₂. Cell lines were routinely checked for the presence of mycoplasma.

To prepare macrophage differentiation medium (DM), Roswell Park Memorial Institute (RPMI) 1640 medium was supplemented with 10% heat-inactivated FBS, 0.1 mM P/S, 25 mM HEPES pH 7.4 and with 20% of L929 conditioned medium (L929-CM), as a source of M‐CSF.

To prepare L929-CM, cells were grown in complete DMEM (10% heat-inactivated FBS, 3.97 mM glutamine, 1 mM sodium pyruvate and 0.1 mM P/S) that was replaced at confluence. After 10 days the medium was collected, centrifuged (750 g, 10 min) and the supernatant was filtered (0.22 μm) and stored at − 80 °C.

To obtain MPNST-CM, 6 × 10⁵ MPNST cells were grown in a 100 mm dish with 10 mL complete DMEM (10% heat-inactivated FBS, 3.97 mM glutamine, 1 mM sodium pyruvate and 0.1 mM P/S). After 4 days, the medium was collected as above and immediately used to treat macrophages.

### Animals

All animal experiments were performed in compliance with applicable international (EU Directive 2010/63) and national (e.g., Italian law D.L. 26/2014) laws, implementing the Directive of ethical committees and the Italian Ministry of Health branch dealing with animal welfare. All experiments were approved by the animal welfare committee (OPBA – Organismo Preposto per il Benessere Animale) from the University of Padova. In this study, we utilized age-matched female C57BL/6 mice, both wild-type (WT) and TRAP1-deficient, approximately 4 months old.

### Macrophage Preparation and treatment

Bone marrow cells were obtained following standard protocols [31]. Briefly, bone marrow cells were collected by flushing the femurs and tibias of WT and TRAP1 KO C57BL/6 female age-matched mice (3–4 months) with complete RPMI using a 25 g needle. The suspension was filtered (cell strainer 40 μm) and centrifuged at 400 *g* for 5 min. After treatment with red blood cell lysis buffer (Sigma-Aldrich) for 10 min at RT, the suspension was neutralized with complete RPMI, centrifuged at 400 *g* for 5 min and cells were resuspended in 10 mL macrophage DM, seeded at an 8 × 10⁵ cell density in a 100 mm low adhesion Petri dish with 10 mL macrophage DM, and incubated at 37 °C in a humidified atmosphere containing 5% CO₂. After 4 days, 5 mL of fresh macrophage DM were added to each Petri dish and cells were kept in culture for 3 additional days when the Mφ macrophage phenotype was assessed.

For the subsequent M2 polarization, Mφ macrophages were treated with 10 ng/mL of IL-4 (ImmunoTools) for 24 h in complete RPMI, and incubated at 37 °C in a humidified atmosphere containing 5% CO₂.

Mφ and M2 macrophages were then exposed to 10 mL of MPNST-CM for 48 h. After the initial 24 h, the media were replaced with fresh MPNST-CM.

To assess the effects of DMS and DM-α-KG, Mφ and M2 macrophages were treated with either 1 mM DMS (SAFC, W239607K) or 1 mM DM-α-KG (Sigma Aldrich, 349631-5G), administered during the two consecutive 24 h exposure of macrophages to the MPNST-CM.

### RNA extraction and quantitative real time reverse transcription PCR (qRT-PCR)

Total RNA was extracted using TRIzol reagent (Thermo Fisher Scientific) and used to synthesize cDNA with the SuperScript III First-Strand Synthesis System (Thermo Fisher Scientific) according to the manufacturer’s instructions. qRT-PCR was performed with the Biorad qRT-PCR machine using SYBR Green (Thermo Fisher Scientific). All reactions were performed on at least 3 biological replicates and the values expressed as fold increase in mRNA levels relative to the housekeeping gene mouse P0 ribosomal protein (*Prlp0*). Absolute values of Ct are normalized against *Prlp0* and fold changes (FC) are calculated using the 2- ΔΔCT method. qRT-PCR primers were designed using primer designing tools from NCBI, NIH and were calibrated before use. Primers are listed in Table 1.

**Table 1:** RT-qPCR primers

GENE	PRIMER
ARG-1	F: 5’- CCACAGTCTGGCAGTTGGAAG R: 5’- GGTTGTCAGGGGAGTGTTGATG
ARG-2	F: 5’ - CTTGCGTCCTGACGAGATCC R: 5’- CCACTCCTAGCTTCTTCTGTCC
CD206	F: 5′- GAGCCCACAACAACTCCTGA R: 5′- TCGCCAGCTCTCCACCTATA
CD163	F: 5′- CAGACTGGTTGGAGGAGAAATC R: 5′- TGACTT GTCTCTGGAAGCTG
CD64	F: 5′- GCTGAGAGCATCCGTGTCAT R: 5′- AAGTGAAGCTGTAAGCCGGG
MGL-1	F: 5’- CAGAATCGCTTAGCCAATGTGG R: 5’- TCCCAGTCCGTGTCCGAAC
VEGF-A	F: 5’- CCACGACAGAAGGAGAGCAGAAGTCC R: 5’- CGTTACAGCAGCCTGCACAGCG
PRLP0	F: 5’- TCACTGTGCCAGCTCAGAAC R: 5’- CTCCCACCTTGTCTCCAGTC
IL-1β	F: 5’- TGGCAACTGTTCCTG R: 5’- GGAAGCAGCCCTTCATCTTT
TNF-α	F: 5’- GCCTCTTCTCATTCCTGCTT R: 5’- TGGGAACTTCTCATCCCTTTG
iNOS	F: 5’ - GCACATTTGGGAATGGAGACTG R: 5’- GGCCAAACACAGCATACCTGA
MMP-9	F: 5’ - GCTCCTGGCTCTCCTGGCTT R: 5’- GTCCCACCTGAGGCCTTTGA
HIF-1α	F: 5’ - AATACATTTTCTCTGCCAGTTTTCTG R: 5’- TTGCTGCATCTCTAGACTTTTCTTTT

### Western immunoblots

To assess protein expression, cells were lysed at 4 °C in a buffer composed by 150 mM NaCl, 20 mM Tris-HCl pH 7.4, 5 mM EDTA, 10% glycerol, 1% Triton X-100, in the presence of phosphatase (Sigma-Aldrich) and protease inhibitors (Sigma-Aldrich). Lysates were then cleared by centrifugation (18000 *g* for 30 min at 4 °C) and proteins were quantified using a BCA Protein Assay Kit (Pierce BCA Protein Assay kit, Thermo Fisher Scientific). For immunoblot analyses, protein extracts were separated in reducing conditions on SDS-PAGE and transferred onto nitrocellulose membranes (Amersham Protran, Sigma-Aldrich) following standard methods. Primary antibodies were incubated for 16 h at 4 °C, and horseradish peroxidase-conjugated secondary antibodies were added for 1 h at RT. Proteins were visualized by enhanced chemiluminescence (UVITEC, Eppendorf). Antibodies are listed in Table 2.

**Table 2:** Primary antibodies for Western blot

Primary antibody	Host	Working dilution	Brand	Catalog
IBA-1	Rabbit	1:1000	Cell Signaling Technology	17,198
TRAP1	Mouse	1:1000	BD Biosciences	612,344
CD206	Rabbit	1:1000	Invitrogen	PA5-114370
ARG-1	Rabbit	1:1000	Cell Signaling Technology	93,668 S
ARG-2	Rabbit	1:1000	Abcam	ab264066
GS	Rabbit	1:5000	GeneTex	GTX109121
MMP-9	Rabbit	1:500	Invitrogen	PA5-27191
HIF-1α	Rabbit	1:500	Novus Biologicals	NB100-449
β-Actin	Mouse	1:5000	Santa Cruz Biotechnology	sc-47,778
β-Tubulin	Rabbit	1:1000	Santa Cruz Biotechnology	sc-9104

### RNA sequencing processing and differential gene expression analysis

Quant Seq 3’ mRNA-seq Library Prep kit (Lexogen) was used for library construction by oligodT priming. The primer already contained Illumina-compatible linker sequences. After first strand synthesis the RNA was removed and second strand synthesis was initiated by random priming and a DNA polymerase. The random primer also contained Illumina-compatible linker sequences. Second strand synthesis was followed by a magnetic bead-based purification step. The library was then amplified, introducing the sequences required for cluster generation. External barcodes were introduced during the PCR amplification step. Library quantification was performed by fluorometer (Qubit) and bioanalyzer (Agilent).

QuantSeq Forward contained the Read 1 linker sequence in the second strand synthesis primer, hence NGS reads were generated towards the poly(A) tail and directly corresponded to the mRNA sequence. QuantSeq FWD maintained strand-specificity and allowed mapping of reads to their corresponding strand on the genome, enabling the discovery and quantification of antisense transcripts and overlapping genes.

Sequencing was performed on NextSeq500 ILLUMINA instrument to produce at least 10 million of reads (75 bp SE) for sample. Raw reads were preprocessed using Trimmomatic [38], (10.1093/bioinformatics/btu170), removing adapter sequences using the Illuminaclip step and employing a sliding window approach averaged on 4 bases and keeping only sequences with average quality evaluated with phred value > 20. Optimal quality was checked employing the FastQC tool (Andrews, S. (n.d.). FastQC A Quality Control tool for High Throughput Sequence Data (http://www.bioinformatics.babraham.ac.uk/projects/fastqc/). Passing filtered reads were mapped to the mouse reference (mm10) using HISAT2 with default parameters. Mapped reads were filtered excluding unpaired mate/pair reads and reads with a MAPQ quality score < 20 using Samtools [39]. The Sequence Alignment/Map format and SAMtools [39]. Raw counts were obtained from filtered reads using featureCounts [40]. Subsequently, differential expression analysis was performed using [41], retaining only genes with a corrected *p*-value < 0.05. For subsequent graphical purposes, normalized counts were extracted from the DESeq object, as well as variance-stabilized values obtained using the vst command from the DESeq2 package.

Obtained differentially expressed genes were filtered employing a previously published pipeline [32] to identify secreted factors.

Moreover, to explore peculiar features of angiogenesis-related factors, genes were additionally filtered following the Hallmark-Angiogenesis collection [33] available in the MSigDB suite [42, 43].

### Heatmap generation

Heatmaps were obtained using default parameters in the heatmap function from the stats R package (R Core Team (2021). R: A language and environment for statistical computing. R Foundation for Statistical Computing, Vienna, Austria) from the variance-stabilized counts obtained from DESeq2. Values are scaled across rows, and dendrograms show similarities between gene trends.

### Cell colony formation assay

To perform the cell colony formation assay, 1.5 × 10^4^ MPNST cells were seeded in a low attachment 24-wells plate without or with 7.5 × 10^3^ WT or TRAP1 KO Mφ/M2 macrophages, previously exposed or not to MPNST-CM, in DMEM without phenol red (25 mM Glucose, 4 mM Glutamine, 1 mM sodium pyruvate, 0.1 mM penicillin and 2% heat-inactivated FBS), supplemented with 4% Matrigel to a total volume of 700 uL/well. The macrophage pre-conditioning with tumor media consisted of two sequential 24 h exposures to fresh MPNST-CM over two consecutive days. After 4 days of incubation at 37 °C in a humidified atmosphere containing 5% CO₂, colonies were stained by carefully adding a crystal violet solution (0.005% crystal violet in PBS, 61135 Sigma Aldrich) dropwise to the edge of each well. After 4 h at RT, colonies were immediately scanned and analyzed using ImageJ software to measure the parameter “integrated density”.

### Boyden chamber assays

To perform Boyden chamber assays, bone marrow-derived macrophages (BMDMs) were differentiated in vitro for 7 days (Day 0). On Day 7, Mφ macrophages were detached and re-seeded in 24-well plates at a density of 5 × 10³ cells per well. Cells were either left unpolarized (Mφ) or polarized toward the M2 phenotype using IL-4 (10 ng/mL) for 24 h. On Day 8, macrophages were exposed to MPNST-CM for 24 h (first exposure). On Day 9, MPNST-CM was replaced with fresh MPNST-CM for a second 24 h exposure (second exposure). On Day 10, the MPNST-CM was removed and replaced with complete DMEM. Macrophages were cultured for an additional 24 h to allow the secretion of soluble factors into the medium (conditioning phase). On Day 11, MPNST cells were seeded at a density of 2 × 10⁵ cells in complete DMEM in the upper compartment of the 24-wells plate in a transwell insert (8 μm pore size). The lower chamber contained macrophages in their enriched medium. MPNST cells were allowed to migrate toward the macrophage-enriched medium.

MPNST cell migration through the membrane of the transwell insert was determined after 16 h using the staining kit RAL Diff-Quik (Siemens Healthineers) according to the manufacturer’s protocol after removing cells from the upper side of the inserts with a cotton swab. Migrated cancer cells were photographed with a LEICA S9i microscope and quantified using ImageJ software by measuring the area stained by crystal violet from three images per condition. Each experimental condition was repeated at least in three replicates.

### Endothelial cell tube formation assays

For the endothelial cell tube formation assay, 8 × 10³ murine SV40-transformed endothelial cells (SVECs) were seeded in 20 µL of complete DMEM per well on a 96-well plate pre-coated with growth factor-reduced basement membrane matrix (Matrigel, Corning, 356231). After 1 h of incubation at 37 °C, each well was filled by adding 80 µL of complete DMEM and 200 µL of MCM. After additional 4 h of incubation, tube formation was examined by Leica DMI4000B inverted microscope. The length of the tubes and the total number of branching points were quantified in triplicate on the entire well surface using ImageJ software.

### In vivo angiogenesis assay and histological analysis

For the in vivo angiogenesis assay, WT and TRAP1 KO macrophages exposed to MPNST-CM were gently detached in cold PBS for 5–10 min, collected by centrifugation at 100 *g* for 8 min and resuspended in complete RPMI medium. Thereafter, 5 × 10⁵ cells were mixed with 500 µL of cold growth factor-reduced basement membrane matrix (Corning, 356231) on ice and injected subcutaneously into the ventral area of 6-week-old nude athymic female mice. All plugs were retrieved after 14 days, fixed in paraformaldehyde (PFA, Sigma-Aldrich), embedded in paraffin and sectioned at a thickness of 4 μm for histological analysis. After deparaffinization in xylene and rehydration in a series of graded alcohols, sections were stained with Mayer’s hematoxylin and eosin (H&E), and vascular buds and blood vessels were identified and counted in five microscopic fields per slice of each plug at a magnification of 40x with LEICA Flexacam i5. Results represent the average of counts from at least seven plugs per group.

To evaluate the presence of macrophages, antigen retrieval was conducted using EnVision FLEX Target Retrieval Solution, High pH (DM828) for 30 min at 97 °C. A 5% BSA solution in PBS was added for blocking of non-specific bindings. Anti-IBA-1 (019-19741, D.B.A. Italia s.r.l.), anti-iNOS (PA3-030 A, Invitrogen), anti-CD163 (BS-2527R, Invitrogen) and anti-CD206 (PA5-114370, Invitrogen) antibodies were used as markers of pan, M1 and M2 macrophages, respectively. After 24 h of incubation at 4 °C, the EnVision FLEX Peroxidase-Blocking Reagent (DM841) was added for 3 min at RT to block endogenous peroxidases, followed by the secondary antibody for 60 min at RT. Thereafter, sections were stained with the chromogen (DAB kit- EnVision FLEX DAB Dako Omnis, GC806) in a humid chamber for 8 min at RT in the dark, and with Hematoxylin for 1 min at RT (C0303, Diapath SPA). Finally, they were dehydrated and sealed. Wash buffer (DM831, Dako EnVision FLEX) was used for washing in all steps.

### Secretome analyses

Composition of the macrophage secretome was evaluated utilizing the proteome profiler mouse angiogenesis array kit (R&D Systems, ARY015) according to the manufacturer’s instructions. Briefly, samples were mixed with a cocktail of biotinylated detection antibodies and then incubated with the membrane spotted in duplicate with capture antibodies to specific target proteins. Captured proteins are visualized using chemiluminescent detection and the produced signal is proportional to the amount of analyte bound.

### Measurement of ROS

Measurements of macrophage ROS were performed by flow cytometry recordings by incubating cells in complete DMEM media depleted of FBS and in the presence of 2’,7’-Dichlorodihydrofluorescein (H₂-DCF) (Molecular Probes) for 30 min. Data acquisition was performed with BD FACSAria™ II and analysis with FACSDiva software.

### Measurement of intracellular succinate

The steady-state level of intracellular succinate was measured in WT and TRAP1 KO Mφ and M2 macrophages exposed or not to MPNST-CM using the fluorometric succinate assay kit (Sigma Aldrich, MAK335) according to the manufacturers’ protocol.

### Statistical analysis

Data were analyzed and presented as mean ± SD in all figures. Pairs of data groups were analyzed using paired and unpaired two-tailed Student’s *t* tests. In the case of more than two groups, one-way analysis of variance (ANOVA) was performed. Statistical significance was determined using GraphPad Prism (version 5). Results with a *p* value lower than 0.05 were considered significant; *P < 0.05, **P < 0.01; ***P < 0.001, ****P < 0.0001 compared to controls. Each experiment was repeated at least three times.

## Discussion

MPNSTs are highly infiltrated by macrophages [9] with a predominant M2-like phenotype [10–12], whose contribution to cancer growth remains poorly defined. Here we show that MPNST cells secrete factors that lead to acquisition of M2 markers (CD206, CD163, MGL-1, MMP-9, VEGF-A, ARG and GS) by macrophages, enabling their induction of cancer cell growth and motility and of endothelial cell angiogenesis. These data point to the existence of a complex molecular crosstalk among different cell types that form the MPNST microenvironment, shaping its growth potential.

Little is known on the metabolic changes that occur during MPNST onset and progression, and the study of how such rearrangements tune the interactions among its cellular components remains an uncharted territory. We observe that expression of the molecular chaperone TRAP1, a master regulator of mitochondrial metabolic circuitries in different cancer cell types [7, 44, 45], is mandatory for skewing the macrophage phenotype toward a M2-like, pro-neoplastic one. In tumor cells TRAP1 down-regulates SDH activity, installing a pseudo-hypoxic condition sustained by succinate-dependent stabilization of the transcription factor HIF-1α [5, 7]. Similarly, we find that also in macrophages TRAP1 expression increases the intracellular levels of succinate and activates a succinate-dependent transcriptional program orchestrated by HIF-1α. Instead, in the absence of TRAP1, MPNST-conditioned macrophages increase intracellular level of α-KG, a co-factor for the hydroxylation of HIF-1α that primes it for proteasomal degradation [35], thus repressing the transcriptional signature orchestrated by HIF-1α.

TRAP1 interacts with several mitochondrial proteins, encompassing metabolic enzymes, transporters and other chaperones [46, 47]. Therefore, its contribution in determining macrophage functions could be multifaceted and stemming from multiple interactions that eventually drive various biological outputs. Nonetheless, administration of exogenous succinate to macrophages lacking TRAP1 increases the expression of HIF-1α and of a panel of HIF-1α-target genes, enacting their pro-neoplastic phenotype and mimicking the presence of the chaperone. Instead, administration of exogenous α-KG interferes with the M2-like phenotype acquisition of TRAP1-expressing tumor-conditioned macrophages. These observations strongly argue in favor of a primary role exerted by a TRAP1-succinate-HIF-1α signaling pathway in the regulation of macrophage functions. Importantly, TRAP1 expression per se is not sufficient for eliciting these downstream effects, as their induction requires the exposure of TRAP1-expressing macrophages to factors secreted by MPNST cells. This suggests that specific signals must prime TRAP1, possibly eliciting post-translational modifications that are known to tune its activity [5, 48, 49]. Elucidating the signaling pathways enrolled in macrophages by MPNST cell-secreted factors will be important for understanding how TRAP1 regulation drives acquisition of a pro-tumoral phenotype in macrophages.

In the latest years, several studies have started to connect the metabolic reprogramming of macrophages with their phenotypic and functional profile [20, 21]. While key metabolic routes that underpin the dichotomy between M1 and M2 states have been characterized, it remains to be dissected whether and how specific metabolic adaptations sustain the variety of phenotypic differences observed in the populations of tumor-associated macrophages [50]. In this scenario, our data highlight the importance of a rise in intracellular succinate levels for polarizing macrophages towards a pro-tumoral phenotype. Succinate is a well-known oncometabolite, as it can induce both transcriptional and epigenetic changes in tumor cells by modulating the activity of α-KG-dependent dioxygenases [51]. More recently, it has emerged that succinate can also influence the activation of macrophages in a complex and nuanced manner. Extracellular succinate implements an anti-inflammatory transcriptional response in M2 macrophages following a selective interaction with the G coupled receptor SUCNR1 [26], and SUCNR1 engagement by cancer cell-derived succinate initiates a PI3K/HIF-1α signaling pathway, skewing macrophages to a M2-like, pro-tumoral polarization [25]. A rise in intracellular succinate has been reported to occur after macrophage exposure to the bacterial toxin lipopolysaccharide (LPS), a potent inducer of M1 polarization, leading to HIF-1α-mediated production of IL-1β [52]; conversely, succinate accumulation obtained by pharmacological inhibition of SDH skews LPS-induced macrophages toward an anti-inflammatory phenotype [23].

These contrasting results draw an intricate picture, in which it is possible that context-dependent elements, such as the type, duration and intensity of the stimuli that increase intra- or extracellular succinate concentration, determine its biological effects. In our model, the rise in succinate is related to TRAP1, which is known to down-regulate SDH activity without fully inhibiting it. This could allow a dynamic tuning of succinate levels, depending on the concomitant level of TRAP1 induction by the factors released by MPNST cells.

Following exposure to the MPNST-CM, only TRAP1-expressing macrophages increase HIF-1α protein levels, but HIF-1α transcription is induced independently of TRAP1. This is in accord with the reported inhibition by TRAP1 of HIF-1α protein degradation via succinate block of the prolyl-hydroxylase that primes HIF-1α for proteasomal degradation [7]. Thus, it can be envisioned that TRAP1 enables macrophages to install a pro-neoplastic transcriptional program orchestrated by HIF-1α independently of the hypoxic conditions found in the cancer regions furthest from blood vessels. The establishment of this pseudo-hypoxic state by TRAP1 could act as a potent factor to amplify the pro-neoplastic functions of macrophages in the cancer microenvironment.

Our study unveils that this HIF-1α-dependent transcriptional program drives the acquisition of a pro-angiogenic signature, leading to the release of a panel of chemokines, growth factors and metalloproteinases that are reduced or almost absent in TRAP1-deficient cells. Some of these factors are well-known inducers of cell motility, which could explain the pro-migratory effect that MPNST-CM exposed, TRAP1-expressing macrophages exert on MPNST cells, even if it cannot be excluded that other mediators are involved in mastering this process. Indeed, NF1-deficient cells can directly promote tumor angiogenesis, as previously reported [53]. Moreover, MPNST specimens exhibit high expression of angiogenic markers, including VEGF and VEGFR family members, which may contribute to tumor growth via an autocrine loop [54, 55]. In line with this, our proteome profiling confirmed that MPNST-CM contains multiple pro-angiogenic factors. However, in our endothelial cell assay, MPNST-CM alone only marginally induced tube-like structure formation. This apparent discrepancy may arise from the short duration of the assay (4 h), chosen to capture the peak of macrophage-induced angiogenesis, after which tube structures typically retract. Such a limited time frame may be insufficient for MPNST-CM to exert its full pro-angiogenic potential, especially given that the concentration of angiogenic stimuli appears lower compared to MCM.

MPNSTs are characterized by a highly heterotypic microenvironment, where multiple subpopulations of different cell types coexist to form a complex ecosystem. Considering the intricacies of these interactions is a conceptual mainstay required for conceiving strategies aimed at blunting MPNST growth. Our data move in this direction, indicating that a TRAP1-succinate-HIF-1α signaling axis is activated in macrophages by MPNST cells and could contribute to the expansion of such an aggressive malignancy. These observations could disclose the identification of key molecular features and druggable pathways targeting microenvironmental components, allowing the advancement of therapeutic discovery studies.

## Conclusions

Our study revealed the existence of a molecular crosstalk between cancer and immune cells whereby macrophages exposed to factors released by MPNST cells undergo a M2-like polarization featured by pro-tumoral functions. Additionally, we unveiled that both MPNST-released signals and TRAP1 expression by macrophages are essential for their phenotype skewing toward pro-neoplastic features which are mastered by a TRAP1-succinate-HIF-1α signaling axis. These findings shed light on an intercellular interaction that could occur within the MPNST microenvironment and whose elucidation could put forward novel therapeutic opportunities aimed at hampering MPNST growth.

### Limitations of the study

Our conclusions rely mainly on in vitro co-culture studies, which indicate that MPNST cells can instruct macrophages toward an M2-like phenotype with tumor-supporting functions. However, in the complex in vivo MPNST microenvironment, macrophage activation is likely shaped by additional crosstalk mechanisms, integrating signals from diverse cellular sources. Moreover, other cell types in addition to macrophages probably contribute to the array of biological routines, including migration and angiogenesis, that shape MPNST growth.

## Supplementary Information

Below is the link to the electronic supplementary material.

Supplementary Material 1

## Data Availability

The bulk RNA-seq data generated during the current study are available in the Gene Expression Omnibus (GEO) database under accession numbers GSE293735. The corresponding author will provide all the data used in this study upon reasonable request.
