# Supplementary material for: TRAP1 expression elicits pro-tumoral functions in macrophages associated to malignant peripheral nerve sheath tumor cells
Source: J Exp Clin Cancer Res. 2025 Aug 28;44:257. doi: 10.1186/s13046-025-03525-1 (PMC12392640; doi:10.1186/s13046-025-03525-1)
Supplement: Supplementary file 1 — Supplementary Material 1 [file 13046_2025_3525_MOESM1_ESM.docx]

**Supplementary material**

**TRAP1 expression elicits pro-tumoral functions in macrophages associated to malignant peripheral nerve sheath tumor cells**

Francesca Scantamburlo^1,2^, Alessia Rubini^1^, Margherita Toffanin^2^, Maria Egle Castorina^1,2^, Francesco Ciscato^2^, Sofia Tomasoni^3^, Paolo Finotti^3^, Ranieri Verin^3,4^, Valentina Zappulli^3^, Marco Fantuz^5,6^, Camilla Bean^7^, Andrea Rasola^1*^, Ionica Masgras^2*^

^1^Department of Biomedical Sciences, University of Padova, via Ugo Bassi 58/B, 35131, Padova, Italy

^2^Institute of Neuroscience, National Research Council, via Ugo Bassi 58/B, 35131, Padova, Italy

^3^Department of Comparative Biomedicine and Food Science, University of Padova, viale dell’Università 16, 35020, Legnaro (PD), Italy

^4^Department of Veterinary Sciences, University of Pisa, viale delle Piagge 2, 56124, Pisa, Italy

^5^Department of Biology, University of Padova, via Ugo Bassi 58/B, 35131, Padova, Italy

^6^Veneto Institute of Molecular Medicine, via G. Orus 2, 35129, Padova, Italy

^7^Department of Medicine, University of Udine, P.le Kolbe 3, 33100, Udine, Italy

*corresponding Authors

**Supplementary Figure 1. Protocol of BMDM isolation and of Mφ/M2 polarization.**


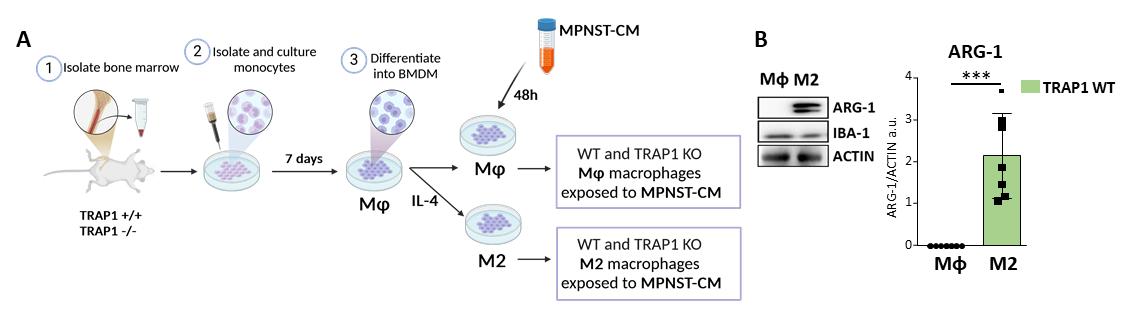


(A) Workflow for BMDM isolation and Mφ/M2polarization. (B) Western blot analysis of the expression of the pan macrophage marker IBA-1 and of the specific M2 marker ARG-1. Actin was used as a loading control. Data are expressed as mean±SD of at least three independent experiments. *p < 0.05, **p < 0.01; ***p < 0.001, ****p < 0.0001 with two-tailed Student’s *t* test.

**Supplementary Figure 2.** **CM of NPCIS and cisMPNST cells induce M2-like markers in TRAP1-expressing macrophages.**

**
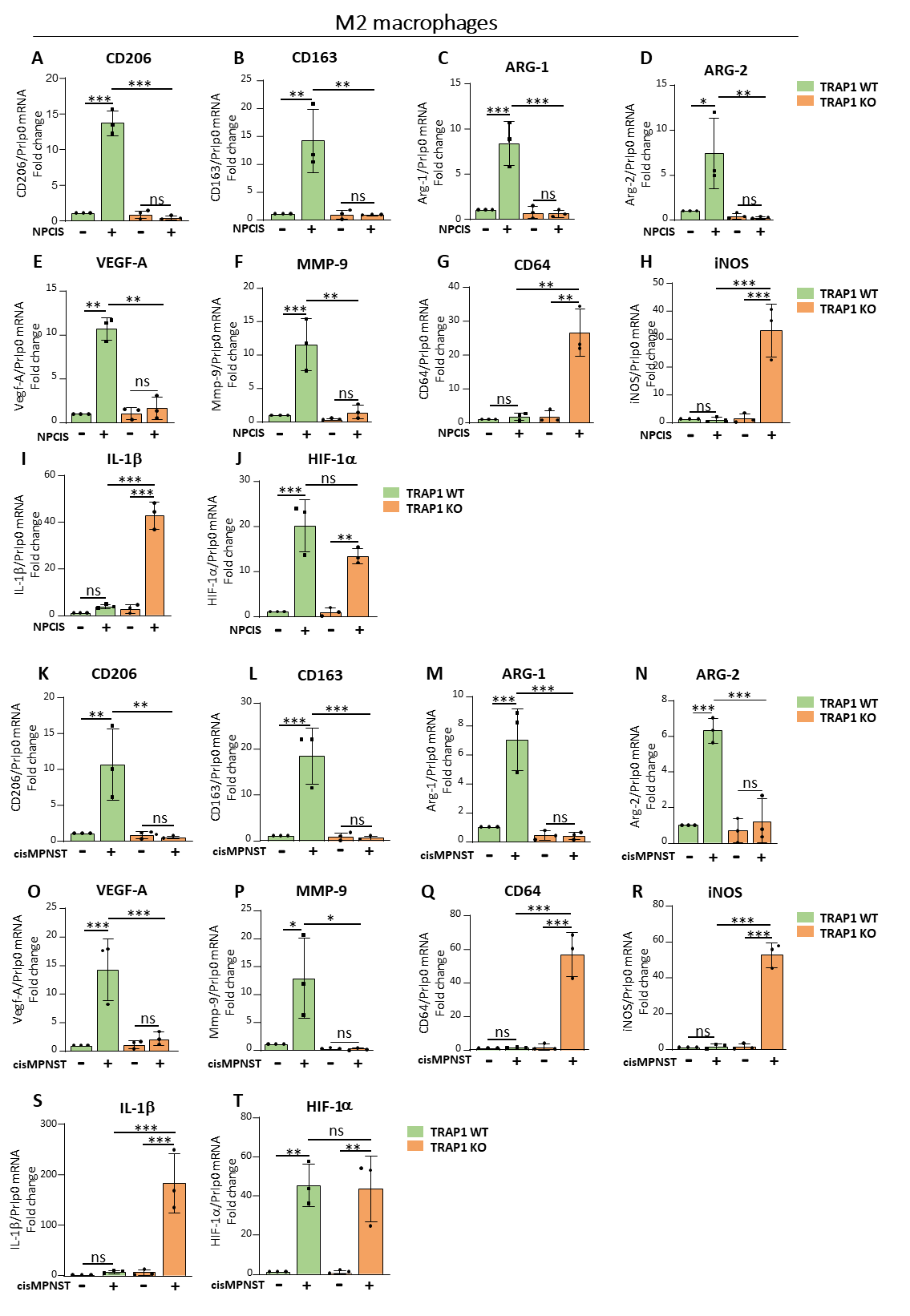
**

RT-qPCR analysis of **(A, K)** *CD206*, **(B, L)** *CD163,* **(C, M)** *Arg-1*, **(D, N)** *Arg-2*, **(E, Q)** *Vegf-A,* **(F, P)** *Mmp-9* **(G, Q)** *CD64*,**(H, R)** *iNos*, **(I, S)** *Il-1β * and **(J, T)** *HIF-1α* mRNA levels in M2 macrophages exposed to CM from NPCIS (**A-J**) or cisMPNST (**K-T**) cells.

mRNA expression levels were normalized *vs*. *Prlp0* and FC (fold change) was expressed as mean±SD of at least three independent experiments. *p < 0.05, **p < 0.01; ***p < 0.001, ****p < 0.0001 with one-way ANOVA test.

**Supplementary Figure 3.** **TRAP1-expressing macrophages exposed to CM from NPCIS and cisMPNST cells display pro-tumoral features.**

**
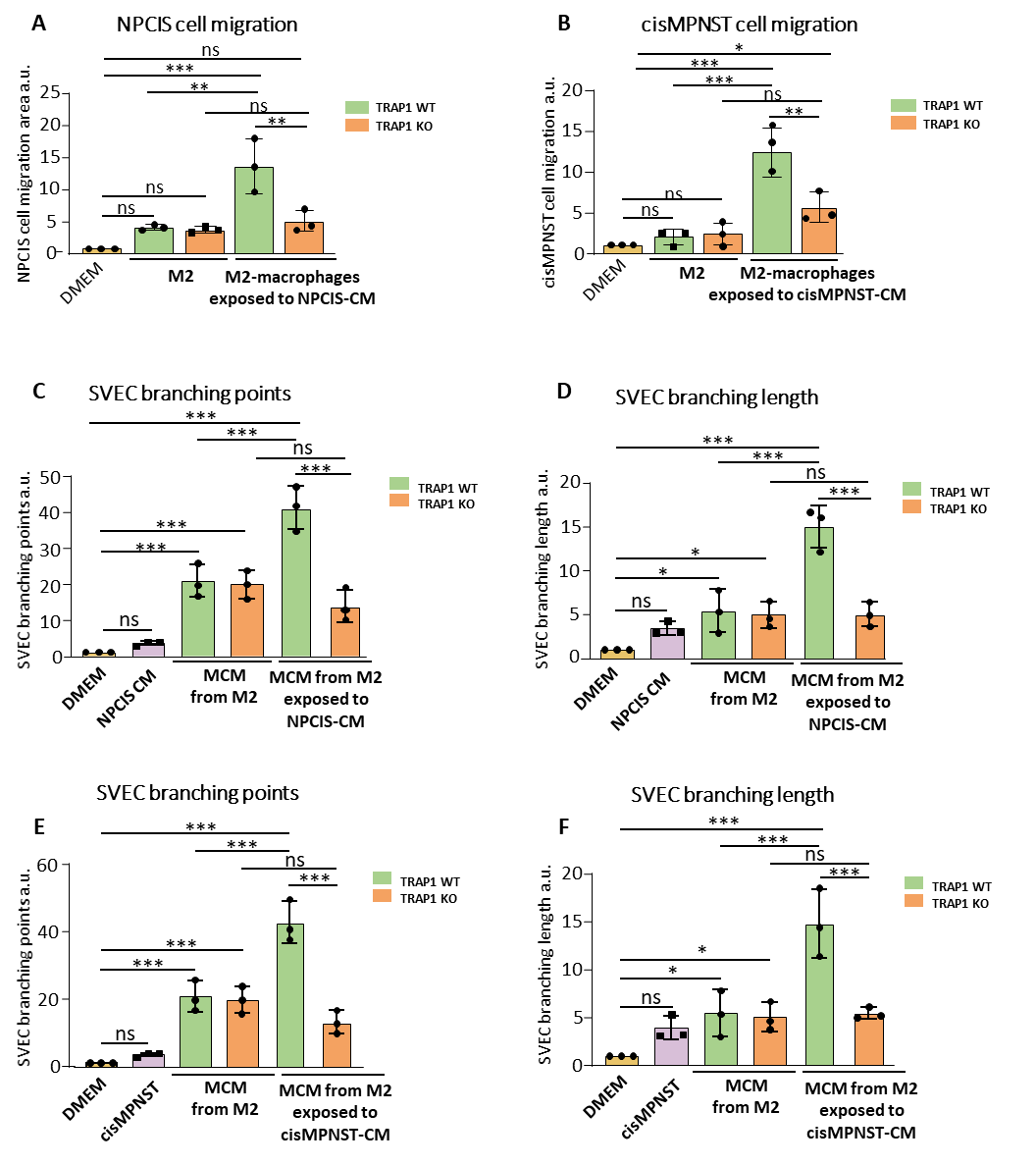
**

Quantification of migration after co-culturing NPCIS (**A**) and cisMPNST (**B**) cells and M2 macrophages; experimental conditions are as in Figure 3E-G.

Quantification of branching points (**C, E**) and branching length (**D, F**) of SVEC cells exposed to DMEM (negative control), to the cancer cell CM and to the MCM obtained from M2 cells, or M2 cells previously exposed to NPCIS-CM (**C-D**) or cisMPNST-CM (**E-F**). Experimental conditions are as in Figure 4.

Data are reported as the FC mean±SD of at least three independent experiments and normalized *vs*. the control. *p< 0.05, **p< 0.01; ***p< 0.001, ****p< 0.0001 with a one-way ANOVA test.

**Supplementary Figure 4.** **TRAP1 expression in MPNST-conditioned macrophages sustains *in vivo* angiogenesis.**
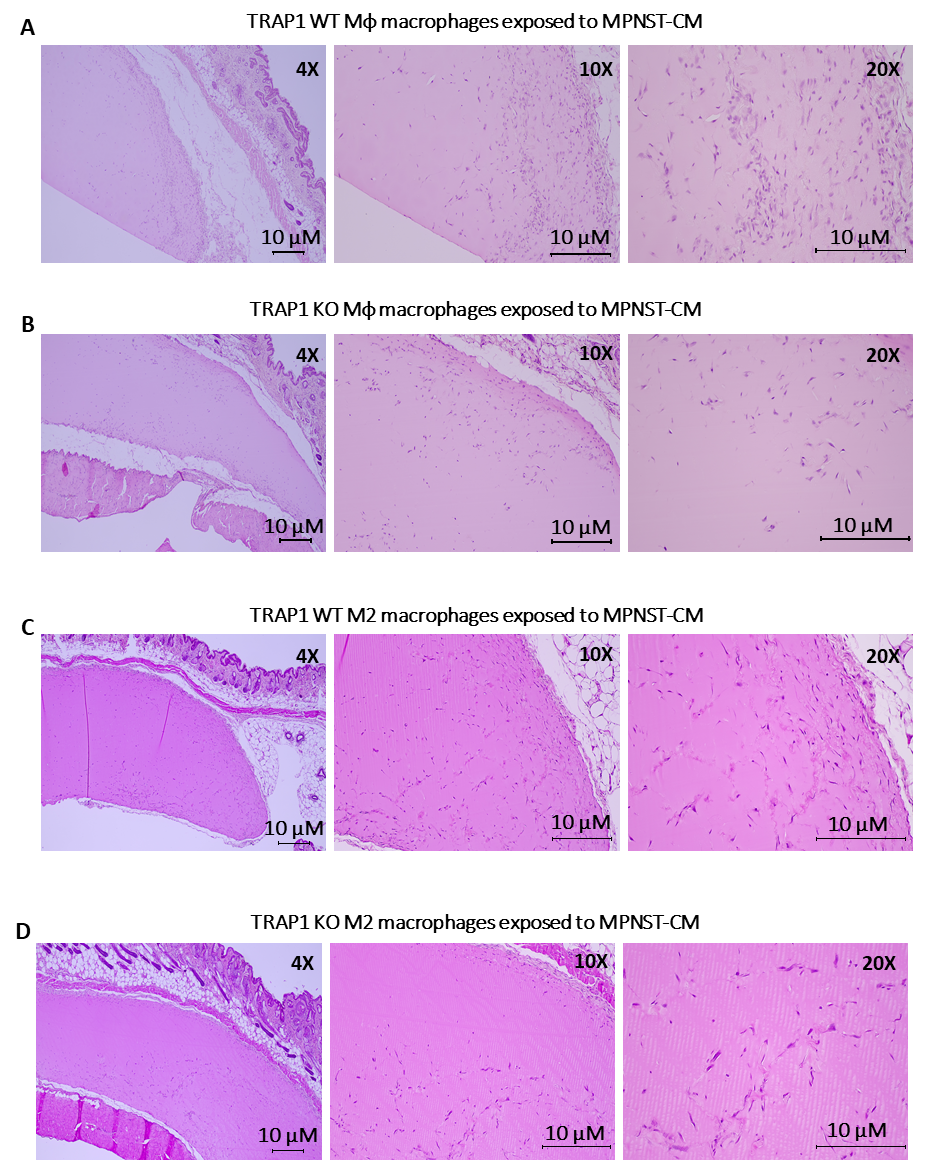


Hematoxylin and eosin staining (H&E) images of Matrigel plugs injected with either MPNST-conditioned Mφ-macrophages WT **(A)** and TRAP1 KO **(B)** or MPNST-conditioned M2 macrophages WT **(C)** or TRAP1 KO **(D)** *in vivo*.

**Supplementary Figure 5. TRAP1 expression increases macrophage number in an *in vivo* angiogenesis experiment.**

**
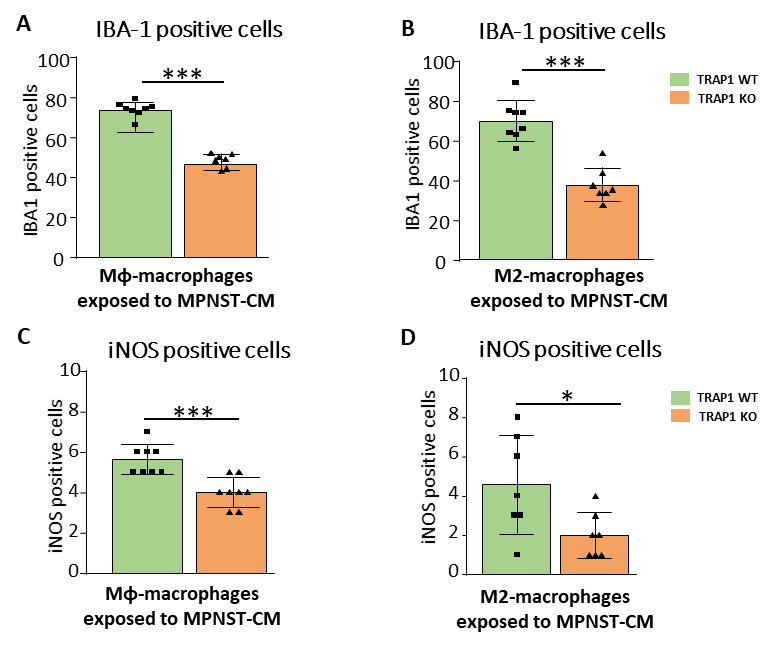
**

(**A-B**) Quantification of cells positive for IBA-1 (**A, B**) and iNOS (**C, D**) in Mφ (**A, C**) and M2 (**B, D**) macrophages exposed to MPNST-CM by IHC inspections of Matrigel plugs. Cells were counted in 10 fields of Matrigel plugs at a 40X magnification. Absolute cell numbers are reported as mean±SD of at least five experiments. *p< 0.05, **p< 0.01; ***p< 0.001, ****p< 0.0001 with a one-way ANOVA test.

**Supplementary Figure 6. TRAP1 expression does not change intracellular ROS levels in macrophages.**
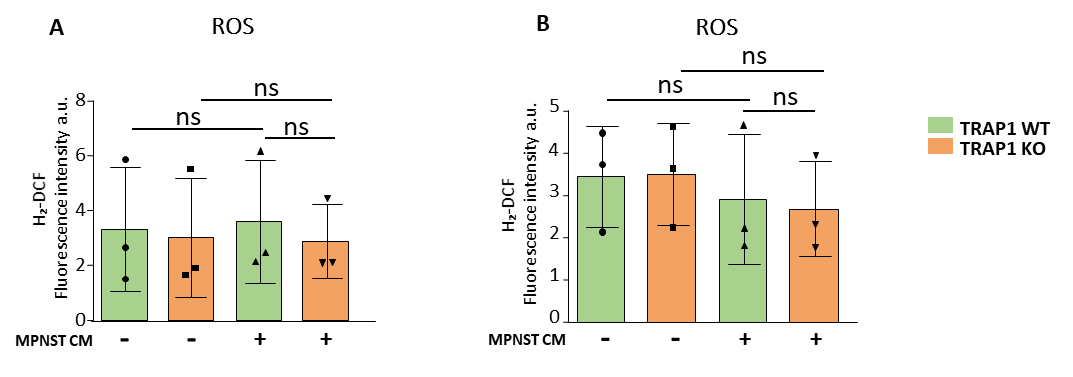


(**A, B**) Quantification of ROS levels with the probe dichlorodihydrofluorescein (H₂-DCF) in TRAP1 WT and KO Mφ (**A**) and M2(**B**) macrophages exposed or not to MPNST-CM. Data are reported as the mean±SD of three independent experiments.

**Supplementary Figure 7. TRAP1-deficient macrophages accumulate α-KG following exposure to MPNST-CM and exogenous α-KG represses the expression of HIF-1α-dependent M2-like markers.**

**
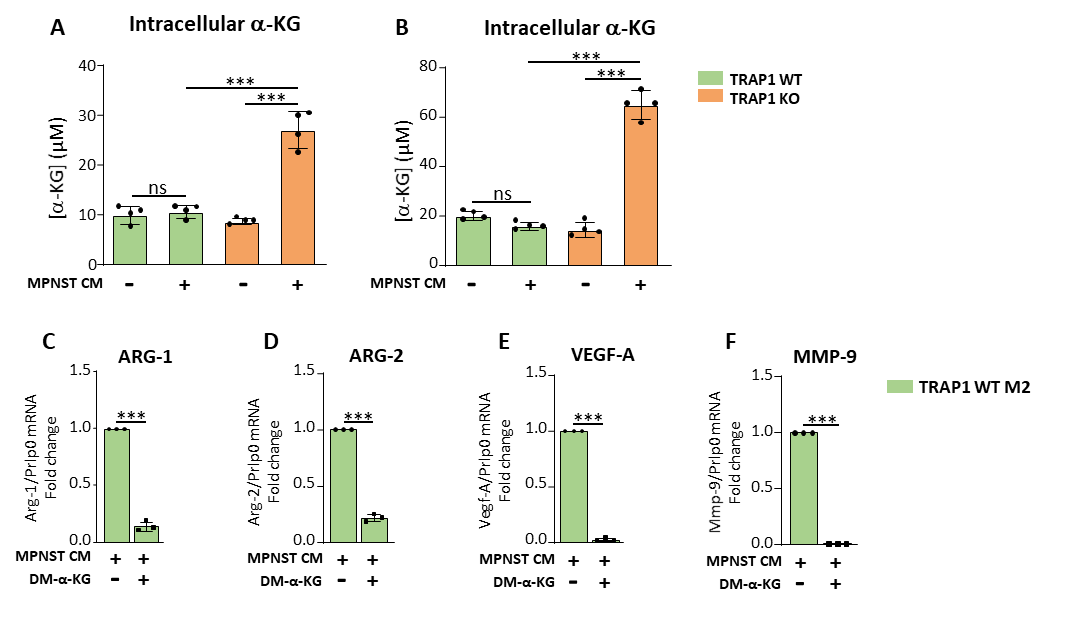
**

(**A, B**) Quantification of intracellular α-KG levels in Mφ (**A**) or M2 (**B**) macrophages. Data are reported as mean±SD of four independent experiments.

(**C-F**) RT-qPCR analysis of (**C**) *Arg-1*, (**D**) *Arg-2*, (**E**) *Vegf-A* and (**F**) *Mmp-9* mRNA levels in TRAP1 WT M2 macrophages cultured in MPNST-CM with or without 1 mM DM-α-KG. FC are reported as mean±SD of at least three independent experiments. *p < 0.05, **p < 0.01; ***p < 0.001, ****p < 0.0001 with one-way ANOVA test.
